# Targeting intracellular nontuberculous mycobacteria and *M. tuberculosis* with a bactericidal enzymatic cocktail

**DOI:** 10.1128/spectrum.03534-23

**Published:** 2024-03-27

**Authors:** Helen P. Bartlett, Clinton C. Dawson, Cody M. Glickman, David W. Osborn, Christopher R. Evans, Benjamin J. Garcia, Lauren C. Frost, Jason E. Cummings, Nicholas Whittel, Richard A. Slayden, Jason W. Holder

**Affiliations:** 1 Endolytix Technology Inc., Beverly, Massachusetts, USA; 2 Department of Microbiology, Immunology, and Pathology, Colorado State University, Fort Collins, Colorado, USA; University of Nebraska Medical Center, Omaha, Nebraska, USA

**Keywords:** NTM, AMR, enzybiotic, liposome, macrophage, capsule, mycomembrane, peptidoglycan, arabinogalactan, phagolysosome, mycobacteriophage, LysA, LysB

## Abstract

To address intracellular mycobacterial infections, we developed a cocktail of four enzymes that catalytically attack three layers of the mycobacterial envelope. This cocktail is delivered to macrophages, through a targeted liposome presented here as ENTX_001. Endolytix Cocktail 1 (EC1) leverages mycobacteriophage lysin enzymes LysA and LysB, while also including α-amylase and isoamylase for degradation of the mycobacterial envelope from outside of the cell. The LysA family of proteins from mycobacteriophages has been shown to cleave the peptidoglycan layer, whereas LysB is an esterase that hydrolyzes the linkage between arabinogalactan and mycolic acids of the mycomembrane. The challenge of gaining access to the substrates of LysA and LysB provided exogenously was addressed by adding amylase enzymes that degrade the extracellular capsule shown to be present in *Mycobacterium tuberculosis*. This enzybiotic approach avoids antimicrobial resistance, specific receptor-mediated binding, and intracellular DNA surveillance pathways that limit many bacteriophage applications. We show this cocktail of enzymes is bactericidal *in vitro* against both rapid- and slow-growing nontuberculous mycobacteria (NTM) as well as *M. tuberculosis* strains. The EC1 cocktail shows superior killing activity when compared to previously characterized LysB alone. EC1 is also powerfully synergistic with standard-of-care antibiotics. In addition to *in vitro* killing of NTM, ENTX_001 demonstrates the rescue of infected macrophages from necrotic death by *Mycobacteroides abscessus* and *Mycobacterium avium*. Here, we demonstrate shredding of mycobacterial cells by EC1 into cellular debris as a mechanism of bactericide.

**IMPORTANCE:**

The world needs entirely new forms of antibiotics as resistance to chemical antibiotics is a critical problem facing society. We addressed this need by developing a targeted enzyme therapy for a broad range of species and strains within mycobacteria and highly related genera including nontuberculous mycobacteria such as *Mycobacteroides abscessus*, *Mycobacterium avium*, *Mycobacterium intracellulare,* as well as *Mycobacterium tuberculosis*. One advantage of this approach is the ability to drive our lytic enzymes through encapsulation into macrophage-targeted liposomes resulting in attack of mycobacteria in the cells that harbor them where they hide from the adaptive immune system and grow. Furthermore, this approach shreds mycobacteria independent of cell physiology as the drug targets the mycobacterial envelope while sidestepping the host range limitations observed with phage therapy and resistance to chemical antibiotics.

## INTRODUCTION

Nontuberculous mycobacteria (NTM) are opportunistic pathogens derived from the environment that are phylogenetically and clinically related to *Mycobacterium tuberculosis* (TB) with similar infection sites primarily in the lung and skin, and with dissemination of mycobacteria in late-stage disease or immuno-compromised patients. The prevalence of diagnosed NTM infections in the USA alone is projected at over 150,000 cases with the incidence of new infections increasing by 8.2% annually (1, 2), whereas according to the World Health Organization, 10 million people per year become ill with TB and 1.5 million die from it. Mycobacteria possess unique resistances to antibiotics in part due to a complicated cell-surface envelope that contains multiple layers: from inside out plasma membrane, peptidoglycan (PG), arabinogalactan (AGL), mycolic acid layer (MA), and capsule layer (CL) (3, 4). Furthermore, pathogenic mycobacteria are known to take up residence within host cells, primarily macrophages (5). These intracellular mycobacteria make drug targeting a difficult obstacle to overcome due to inaccessibility. For this reason, it is often difficult to completely cure these intracellular infections, resulting in years of treatment, testing, and a high rate of NTM reemergence post-therapy (6).

Currently, patients with NTM infections face poor choices for treatment often requiring years of treatment with multiple small molecule antibiotic cocktails. The usage of a cocktail of different antibiotics is the defined standard of care (SoC) by the Infectious Disease Society of America (IDSA) for *Mycobacterium tuberculosis and Mycobacterium avium* complex (MAC). While variations of the IDSA antibiotic cocktail protocols are used for *Mycobacteroides abscessus* infections, there is no clinically defined protocol for the rapid-growing species.

Antimicrobial resistance (AMR) poses one of the greatest threats to global health. Estimates suggest antibiotic resistance burdens the U.S. healthcare system at a cost of more than $5 billion annually and results in the death of more than 35,000 people (7). Mycobacterial resistance to frontline antibiotic therapies requires secondary and even tertiary therapy regimens that extend hospital stays and recuperation time (8). Due to the prolonged exposure and associated toxicity of antibiotics used to treat mycobacterial infections, drug treatment interruptions are a common occurrence leading to additional costs and increasing the risk for infection relapse (9). Studies estimate NTM infections relapse at a rate between 20% and 44% (6). Though protocols vary, a patient is considered cleared of NTM following between 10 months and 12 months of negative cultures after treatment (10, 11). Taken together, there are critical unmet needs in the treatment of mycobacterial infections.

Endolytix Cocktail 1 (EC1) was designed using four enzymes that catalytically attack three layers of the mycobacterial envelope and comprised LysA, LysB, isoamylase, and α-amylase. LysA, a bacteriophage-encoded protein, represents a functionally linked family of proteins capable of degrading the PG layer during lytic release of progeny phage (12); interestingly, mycobacterial *lys*A genes are highly divergent within the genus suggesting diverse enzymatic activities exist within the family (13). Mycobacterial LysA proteins are distantly related to PlyC, a LysA enzyme that has been used to treat streptococcal infections (14). LysA enzymes have been described as excellent candidates for anti-mycobacterial therapies if limitations in accessing the target peptidoglycan layer are addressed (13, 15).

LysB proteins are exclusive to bacteriophages whose hosts contain an MA layer, as in the Actinomycetia class of gram-positive bacteria. LysB is a serine esterase that has been demonstrated to hydrolyze the linkage between heteropolymer arabinogalactan and mycolic acids of *in vitro* substrates (16), while Gil et al. demonstrated the product release of free mycolic acids from the mycobacterial envelope (17). LysB derived from mycobacteriophage D29 has been characterized genetically as key for release of mycobacteriophage (16). As a therapeutic agent for mycobacterial infections, a previous study showed the ability of LysB to inhibit proliferation of *Mycobacterium ulcerans* infections in mouse footpads and a 20-fold reduction in *M. abscessus* cell numbers was observed over 9 days in an immunocompromised mouse model using aerosolized LysB (18, 19).

The capsule-degrading enzymes, α-amylase and isoamylase, hydrolyze α-1,4 (EC 3.2.1.1) and α-1,6 linked glucose (EC 3.2.1.68) within the capsular polysaccharides, respectively. The reported structure of the polyglucans in the mycobacterial envelope CL consists of heptamer α-1,4-glycan chains linked by a single α-1,6-glycosidic connection (3). Cleaving the capsule polysaccharides exposes the MA layer facilitating access to the target layers for both LysA and LysB through the action of LysB. These enzymatic cleavages of capsule could also serve to destabilize the envelope structure when combined with the other enzyme activities in the cocktail. α-Amylase was previously shown to inhibit growth-dependent luciferase production *in vitro* of *M. tuberculosis* in combination with Ms6 LysB but was largely dependent on the detergent Tween 80 (20).

Mycobacteria are capable of targeting and surviving within macrophages by arresting the progression of a phagosome into a phagolysosome (21, 22). Mycobacteria such as *M. tuberculosis* and *M. avium* have been shown to grow within the developmentally arrested phagosome leading to host cell death and subsequent uptake of mycobacteria into other macrophages where the same pattern repeats (23). The intracellular lifecycle of mycobacteria presents another major challenge in the treatment of mycobacterial disease, for the bioavailability of drug at the sites of the infection. To address this problem, we introduced macrophage-targeted liposomes as an enzyme delivery vehicle. We assembled poly-proteo-liposomes (PPLs) named ENTX_001 for the efficient uptake and delivery of enzymes (EC1) to the intracellular sites of infection.

Here, we demonstrate a mechanism of action for EC1 that results in mycobacterial cell death by shredding mycobacterial cells into subcellular fragments, i.e., debris. We also demonstrate the ability of ENTX_001 to effectively rescue macrophages from the necrotic phenotype associated with mycobacterial intracellular infected macrophages (IIM) that is enhanced through targeted liposome drug delivery. We present the performance of the cocktail-free enzymes *in vitro* and encapsulated formulation on NTM species *ex vivo* as a proof of principle for a drug-delivered enzymatic cocktail capable of killing *M. abscessus*, *M. intracellulare*, *M. avium*, and *M. tuberculosis*.

## RESULTS

### Design of the EC1

To develop a lytic cocktail for mycobacteria, we used a combination of homology searching and domain identification of mycobacteriophage genomes to discover mycobacterial lytic genes that would attack the mycobacterial envelope at three layers (CA, AGL, and PG). To identify a diverse set of LysA proteins that have been shown to be PG-degrading enzymes, we searched mycobacteriophage genes in PhagesDB resulting in 1,829 candidate *lys*A genes representing 27 unique domain archetypes (Fig. S1). We conducted a protein expression screen of one representative gene in the 27 *lysA* archetypes. We found LysA from the mycobacteriophage Yunkel11 (YP_009954207.1) to be a protein that had the most desirable features of being readily isolable and thermally stable and therefore characterized it further.

After processing 1,651 *lys*B genes in the same manner as *lys*A genes, we identified 14 unique domain archetypes (Fig. S2). We repeated a similar protein expression screen for one representative of each gene family to find the previously studied *lys*B gene encoded by the mycobacteriophage D29 (NP_046827.1) had desirable expression, thermal stability, and enzymatic activity (16).

To degrade the CL containing α-1,4 and α-1,6 linkages within glycan, we searched the CaZy database to identify enzymes with the correct enzymatic activity resulting in identification of α-amylase and isoamylase (24). We both commercially sourced *Bacillus licheniformis* (CAA01355.1) and expressed *Rhizomucor pusillus* α-amylase (AGJ52081.1). Isoamylase was isolated from *Candida glabrata* (CAG59721.1) due to high yields that were attained previously (22). To characterize the stability of EC1, we analyzed all four proteins individually in the EC1 by thermal challenge-coupled spectroscopy in the Uncle instrument (Unchained Labs, Pleasanton, CA) to discover that all four proteins had aggregation temperatures (*T*_agg_) > 45°C and melting temperatures (*T*_*m*_) > 46°C, while as a four-protein cocktail, *T*_agg_ > 47°C and *T*_*m*_ > 53°C. These stability values exceed that of the human body and thereby support further development as a therapeutic.

### The EC1 demonstrates bactericidal effects via shredding of *M. abscessus* cells

**Fig 1:**
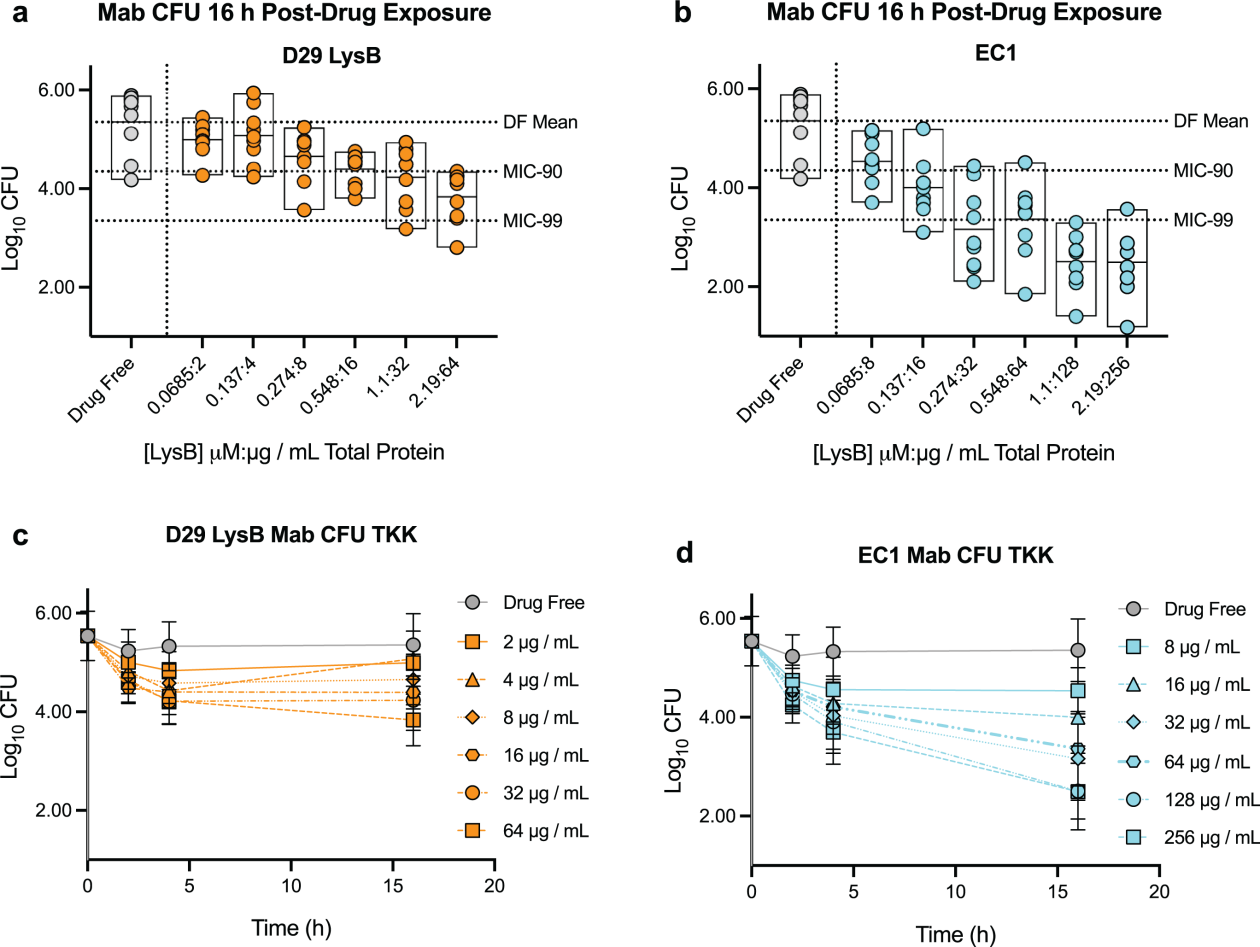
CFU enumeration comparing viability of LysB and EC1 enzybiotics on *M. abscessus*. (a) LysB from mycobacteriophage D29 is titrated in enzymatic reactions with *M. abscessus* for enumeration of CFU. Micromolarity of LysB and microgram per milliliter total protein are both shown on the *x*-axis. (b) Same as panel a for the EC1 enzymatic cocktail. (c) Time-kill kinetics (TKK) were measured via CFU for D29 LysB acting on *M. abscessus* over 16 h. (d) Same as panel c, instead with the EC1 of enzymes.

To evaluate whether the EC1 (LysA, LysB, isoamylase, and α-amylase) cocktail is bactericidal, we first treated 2.5 E5 *M. abscessus* cells with either LysB or EC1 and monitored the quantity of viable cells by enumerating colony forming units (CFU). Equivalent amounts of LysB were compared as the four-protein cocktail was used at 1:1:1:1 mass ratio. Importantly, the killing reactions were done with washed cells in a reaction buffer that lacks Tween 80 and plated on Middlebrook 7H10 oleic acid-albumin-dextrose-catalase (OADC) plates that also lack Tween 80. LysB phenotypes of killing mycobacteria *in vitro* have largely been done in the presence of Tween 80 and shown to have a significant impact on the resulting cell viability (20, 25, 26). In addition, 0.548 µM LysB or 16 µg/mL is able to achieve a 90% reduction MIC_90_ in cell number (Fig. 1a), whereas EC1 can do the same at 0.137 µM [LysB] in a total of 16 µg/mL total protein for the four-protein cocktail, a fourfold reduction in [LysB]. Lys B alone was unable to attain an MIC_99_ under these conditions, whereas EC1 was able to reach that cellular reduction at 0.274 µM LysB and 32 µg/mL in total cocktail protein concentration. A notable difference between LysB alone and with the other components of the EC1 is the plateau in the kinetics of death for LysB alone after 4 h (Fig. 1c), whereas EC1 continued a strong downward trend for concentrations above MIC_90_ (Fig. 1d).

Mycobacteria enter cells through endocytosis and move through the endosomal to lysosomal pathway; however, multiple decades of research indicate that mycobacteria delay and modify normal lysosomal progression (27) resulting in retention in endosomal compartments by inhibiting late endosome fusion to lysosomes (21) and recruitment of acid hydrolases resulting in compartments that prevented acidification below 6.3 for *M. avium* (28). To evaluate whether EC1 had the right pH activity profile, we modified the pH from 7.5 to 6.6 by titrating down glycine concentration in the reaction buffer followed by grow-out in 7H9 media that contains Tween 80 which is used to prevent excessive clumping.

**Fig 2:**
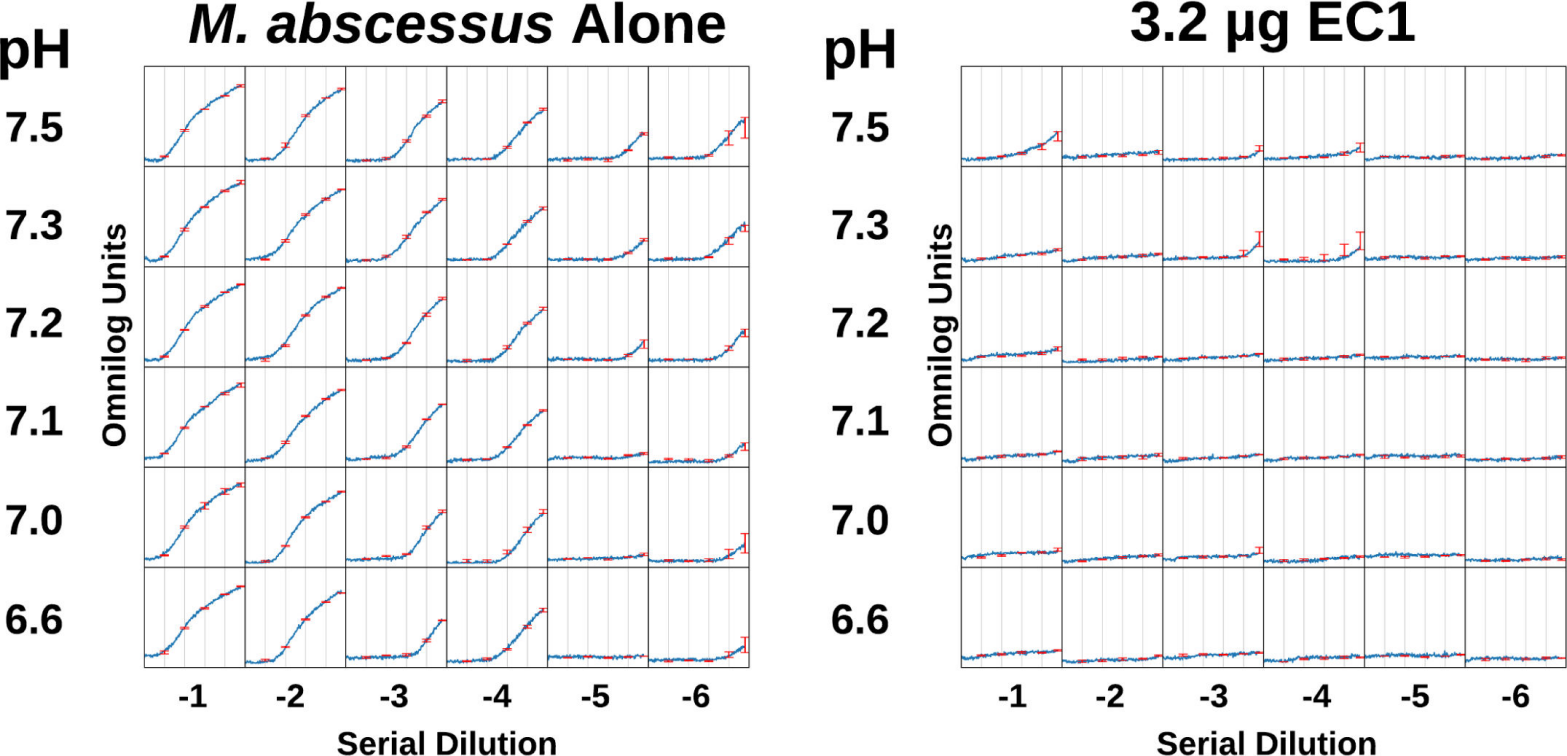
Measurement of the impact of EC1 on growth of *M. abscessus* across a 10-fold dilution series (*x*-axis) while titrating for pH (*y*-axis). *M. abscessus* growth is measured by absorbance with a redox-active dye that absorbs at 590 nm. All conditions are seeded with 1E5 *M. abscessus* cells. Killing reactions are run for 24 h and then serially diluted into media that does not contain drug. The gray vertical lines in the growth curves represent days of growth (total 121 h).

1E5 *M. abscessus* cells was treated with 0.8 µg of each EC1 component (64 µg/mL of total protein in equal mass ratios) for 24 h in reaction buffer then the enzymatic reaction products were serially diluted prior to growth for 5 days in 7H9 media lacking EC1. We evaluated growth with kinetic measurements taken many times each day using an Omnilog (Biolog, Inc.) machine that incubates multiwell plates at 37°C and measures growth optically in place, revealing growth was evident after 2 days in the absence of drug, whereas EC1 was able to entirely prevent growth through five 10-fold dilutions for 5 days of grow-out in the absence of EC1. We found out that EC1 killed bactericidally as a single EC1 treatment eliminated the growth of 1E5 *M. abscessus* cells as grow-out was expected if EC1 was acting statically. By varying the pH from 7.5 to 6.6 with a glycine pH 8.0 titration, we demonstrated that bactericidal activity was improved with lowering of the pH (Fig. 2). Stimulation of enzymatic activity is consistent with the pH enzymatic optima for the capsule-degrading enzymes α-amylase (~4.5) and isoamylase (~7.0) (29, 30). This activity pH range is congruous with the development of phagosome into lysosomes, indicating this enzyme cocktail has the right pH profile for *in vivo* activity.

**Fig 3:**
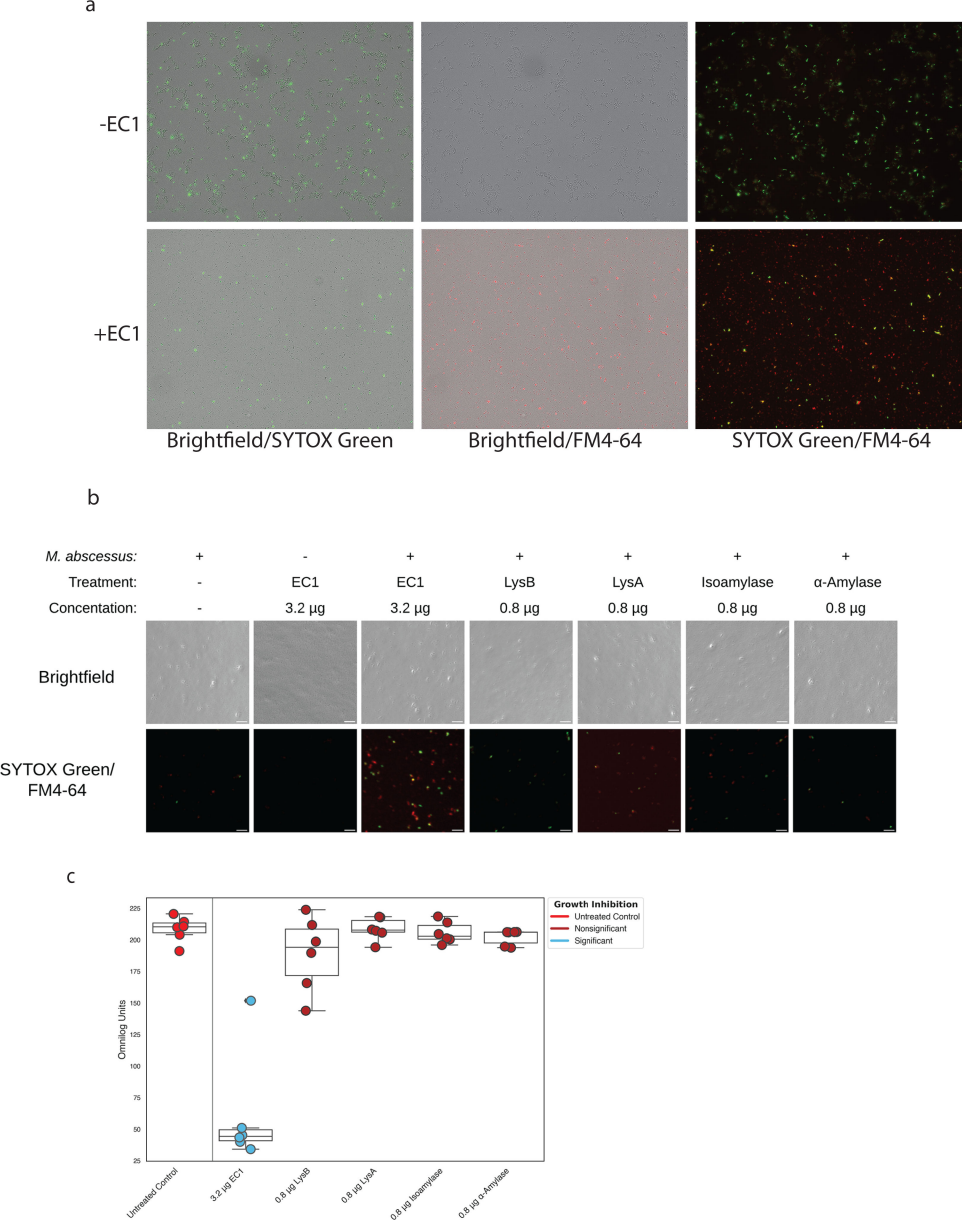
(a) Comparison of EC1-treated *M. abscessus* with dual overlay images of Brightfield/SYTOX Green, Brightfield/FM4-64, and SYTOX Green/FM4-64. Top row is untreated and bottom row is treated with 32 µg/mL of EC1. (b) Brightfield and fluorescence microscopy images of *in vitro* EC1 reactions with *M. abscessus*. The top panel contains brightfield images, whereas the bottom panel contains a merged image of the fluorescence of SYTOX Green that binds DNA and FM4-64 that binds to the outer leaflet of the plasma membrane. The scale bar is 10 µm. (c) Growth data after 5 days using redox-sensitive dye comparing EC1 with the individual components LysB, LysA, isoamylase, and α-amylase. All reactions contained 0.8 µg of each enzyme component.

We further analyzed the bactericidal effects of EC1 using fluorescence microscopy with envelope-limited permeability dyes SYTOX Green for DNA and lipophilic FM4-64 to stain *M. abscessus* after an overnight incubation in enzymes (Fig. 3a). We present light signal overlays for Brightfield and SYTOX Green to demonstrate that in untreated samples, the DNA stained green within some rod shaped cells, due to limited dye uptake by intact cells. The FM4-64 staining demonstrates the effect EC1 has to increase the red signal revealing the many red particles released from *M. abscessus*. The overlay of FM4-64 and SYTOX Green fluorescent signals reveals stark differences between ±EC1 treatments, wherein the EC1 products are clearly separated red and green staining particles. The shredded cellular products of EC1 were generated in reaction buffer at pH 5.3 (Fig. 3a), further supporting the pH requirements for physiologic activity are met by the EC1 enzymatic cocktail.

We compared the EC1 to the individual enzyme components revealing that EC1 was highly effective at stimulating fluorescence via shredding the *M. abscessus* cells into cellular debris that contained either FM4-64 staining lipids or SYTOX Green staining DNA as seen in a merged view of both fluorescent signals (Fig. 3b). Individual fluorescence signals are shown in Fig. S3. All the individual enzyme components had greatly diminished fluorescent signals due to dye inaccessibility to the plasma membrane, triacylglycerols, and genomic DNA (31). We further analyzed the products of these reactions by growth for 121 h at 37°C in the absence of drug that revealed EC1 was effective at inhibiting growth, whereas the other proteins either had no effect or a diminutive growth effect for LysB at the same concentration as in EC1 (Fig. 3c). These results demonstrate that the components of this cocktail act collectively to promote bactericide through a shredding mechanism resulting in cellular debris separating the membranes from the genomic DNA.

**Fig 4:**
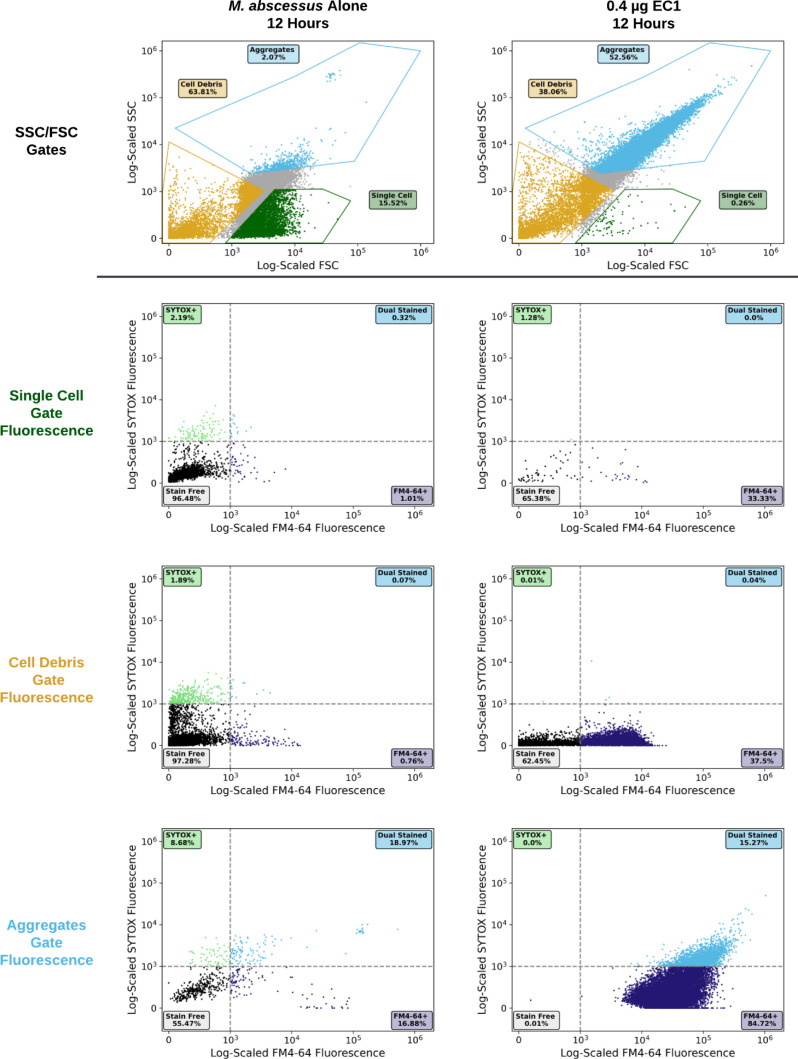
(Top row) Gates were established by the size and features of single cells then the particles resulting from enzymatic digestion by EC1 (right column) as seen in forward- (FSC) and side-scattered (SSC) light, respectively, via flow cytometry. We detected gates that could separate *M*. a*bscessus* populations of single cells, cellular debris, and aggregates after treatment with EC1 *in vitro*. Cells (row 2), cell debris (row 3), and aggregates (row 4) were stained with SYTOX Green DNA Stain and lipophilic FM4-64 stain. The *x-* and *y*-axes represent increasing FM4-64 and SYTOX fluorescence intensity, respectively, in logarithmic scale and range incrementally from 10^3^ to 10^6^.

To further characterize the EC1 enzymatic products on a larger population of mycobacteria, we used flow cytometry on 1E5 *M. abscessus* cells. We established a single cell gate for *M. abscessus* using background buffer normalization then characterized the profile with disaggregated mycobacteria. After 12 h of enzyme treatment of *M. abscessus,* we noted a shift in the population of single cells to two new populations, one being smaller and enriched in cellular debris and a second population that was much larger than single cells and debris. To establish the cellular debris and aggregate gates, we further analyzed the enzymatic products with FM4-64 and SYTOX Green which supported the gate formation of cellular debris as being FM4-64 particles lacking SYTOX staining unlike single cell and aggregate populations. The aggregate population contained both SYTOX Green and FM4-64 staining material and was much larger yet dependent on enzymatic treatment. The observation of aggregate formation is consistent with previous studies that demonstrate these cellular aggregates form with mycobacteria that have been treated with lethal quantities of envelope-acting antibiotics (Fig. 4) (32). Using the established gates, we evaluated the enzymatic degradation of *M. abscessus* using FM4-64 and SYTOX Green fluorescence at 1,2, and 12 h to reveal that the single cell population is depleted while the debris and aggregate populations increase as a function of time. We observe dual-staining debris after 1 h but are mostly gone by 2 h, whereas the bulk of the cellular debris is FM4-64 staining only, indicating the cellular debris lacks genomic DNA. The aggregate population at 1 h is enriched in dual staining material, but by 12 h, the aggregate population is predominantly FM4-64 alone staining consistent with release of genomic DNA from the aggregated material. (Fig. S4). We suggest that, *in vitro,* the larger aggregated material results from hydrophobic interactions resulting from the unpacking of a large amount of hydrophobic material released from the mycomembrane and other lipid compartments. Our flow cytometric analysis confirmed what we observed by growth and microscopy, that EC1 is bactericidal via attacking the mycobacterial envelope and shredding cells down into debris allowing for the separation of genomes from the cells from which they are derived.

### Minimum inhibitory concentrations (MICs) of EC1 alone and in combination with standard-of-care antibiotics

With the demonstration of bactericidal shredding behavior observed with *M. abscessus,* we set up to determine MIC in a standard plate assay according to Clinical and Laboratory Standards Institute (CLSI) standards (33). We expanded our analysis to four species and eight strains from both rapid-growing *M. abscessus* and slow-growing *M. avium*, *M. intracellulare*, and *M. tuberculosis* (Table S1). Our results indicate that EC1 kills broadly across mycobacteria and highly related *Mycobacteroides* (Table 1).

**TABLE 1:** Standard CLSI testing of drugs against *M. abscessu*s (Mabs) 19977 and 103, *M. avium* Mac 101, *M*. *avium* (Mav) 2285R and ECL94, *M intracellulare* (Mint) ECL55, and *M. tuberculosis* (Mtb) H37Rv and TN587^*a*^

MIC (µg/mL)	Mabs 19977	Mabs 103	Mac 101	Mav 2285R	Mav ECL94	Mint ECL55	MIC (µg/mL)	Mtb H37Rv	Mtb TN587
EC1	8.00	4.00	0.50	0.50	10.00	4.00	EC1	32.00	32.00
CLA	0.125	0.125	2.00	1.00	2.00	0.25	INH	0.25	2.00
MOX	1.00	1.00	0.50	2.00	16.00	8.00	RIF	0.25	64.00
FOX	8.00	8.00	8.00	32.00	8.00	1.00	FOX	2.00	16
AMI	4.00	2.00	16.00	16.00	16.00	2.00	AMI	0.25	8.00

^*a*^ The drugs used were EC1, clarithromycin (CL A), moxifloxacin (MOX), cefoxitin (FOX), imipenem (IMI), amikacin (AMI), isoniazid (INH), and rifampicin (RIF).

The efficacy of EC1 was then directly compared against common frontline antibiotic therapies with a set of pathogenic strains. The MIC of EC1 when paired with four SoC drugs was also measured against the same set of NTM and *M. tuberculosis* to evaluate whether they are synergistic, additive, indifferent, or antagonistic in killing mycobacteria. To this end*,* we performed MIC assays with combinations of drugs, revealing dramatic reductions in the required doses of SoC drugs *in vitro* (Table 2). Combination *in vitro* drug data are interpreted though the fractional inhibitory concentration index (FICI) calculation (Fig. S10), indicating no antagonism and that all of the drugs were synergistic for all mycobacterial species tested. Furthermore, all but one strain, *M. tuberculosis* H37Rv, were synergistic, whereas in strain H37Rv, we only detected additive effects.

**TABLE 2:** The MICs of combinations of SoC drugs with EC1 are represented in microgram per milliliter for the SoC drug

MIC (µg/mL)	Mabs 19977	Mabs 103	MAC 101	Mav 2285R	Mav ECL94	Mint ECL55	MIC (µg/mL)	Mtb H37Rv	Mtb TN587
CLA	0.025	0.025	0.1	0.2	0.2	0.05	INH	0.05	0.4
MOX	0.025	0.1	0.05	0.2	0.05	0.05	RIF	0.05	0.4
FOX	0.4	0.1	0.05	0.4	0.05	0.05	FOX	1.6	0.4
AMI	0.025	0.025	0.025	0.2	0.05	0.05	AMI	0.05	0.8

We noted large reductions in the MICs of SoC drugs when combined with EC1 that were species and strain dependent. Combinations of EC1 with amikacin and cefoxitin were notably potent for both NTM in the range of 20- to 640-fold reduction in SoC concentrations required and *M. tuberculosis* in the range of 1.25- to 40-fold reductions (Table 2). We also noted a strong synergy with rifampicin for *M. tuberculosis* ranging from 5- to 160-fold reductions in required SoC drug concentration reflecting in part the highly antibiotic-resistant nature of the *M. tuberculosis* strain TN587 and a powerful demonstration that an enzybiotic approach bypasses AMR.

**Fig 5:**
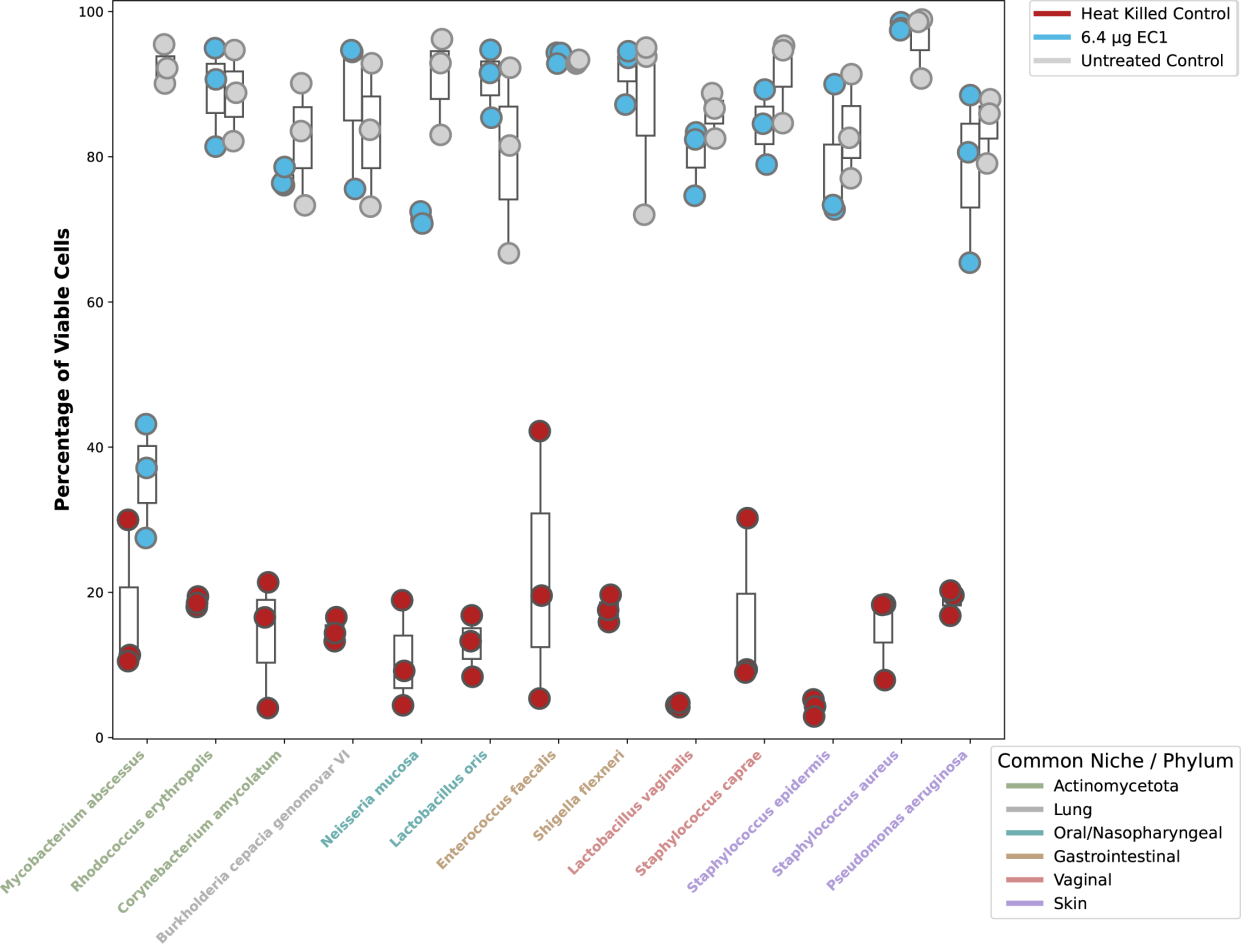
Evaluation of EC1 effects on off-target species was performed with viability dyes SYTO9 and propidium iodide (Molecular Probes) to evaluate untreated strains from that of heat-killed and EC1-treated cells.

Having demonstrated the potency and synergies with four divergent species of mycobacteria and *Mycobacteroides*, we went on to evaluate the effect EC1 has on bacterial species outside of the order of Mycobacteriales. We evaluated 12 off-target species both pathogenic and commensal to interrogate the specificity of EC1. We went further to include some phylogenetic relatives of mycobacteria in the Mycobacteriales order, namely *Rhodococcus erythropolis* and *Corynebacterium amycolatum,* both of which have a related envelope, cell wall, and membrane-integrated biopolymers yet all off-target species tested were not affected by the EC1 (Fig. 5).

### Encapsulation of EC1 enzymes to create ENTX_001 protects infected macrophages from necrotic death *ex vivo*

**Fig 6:**
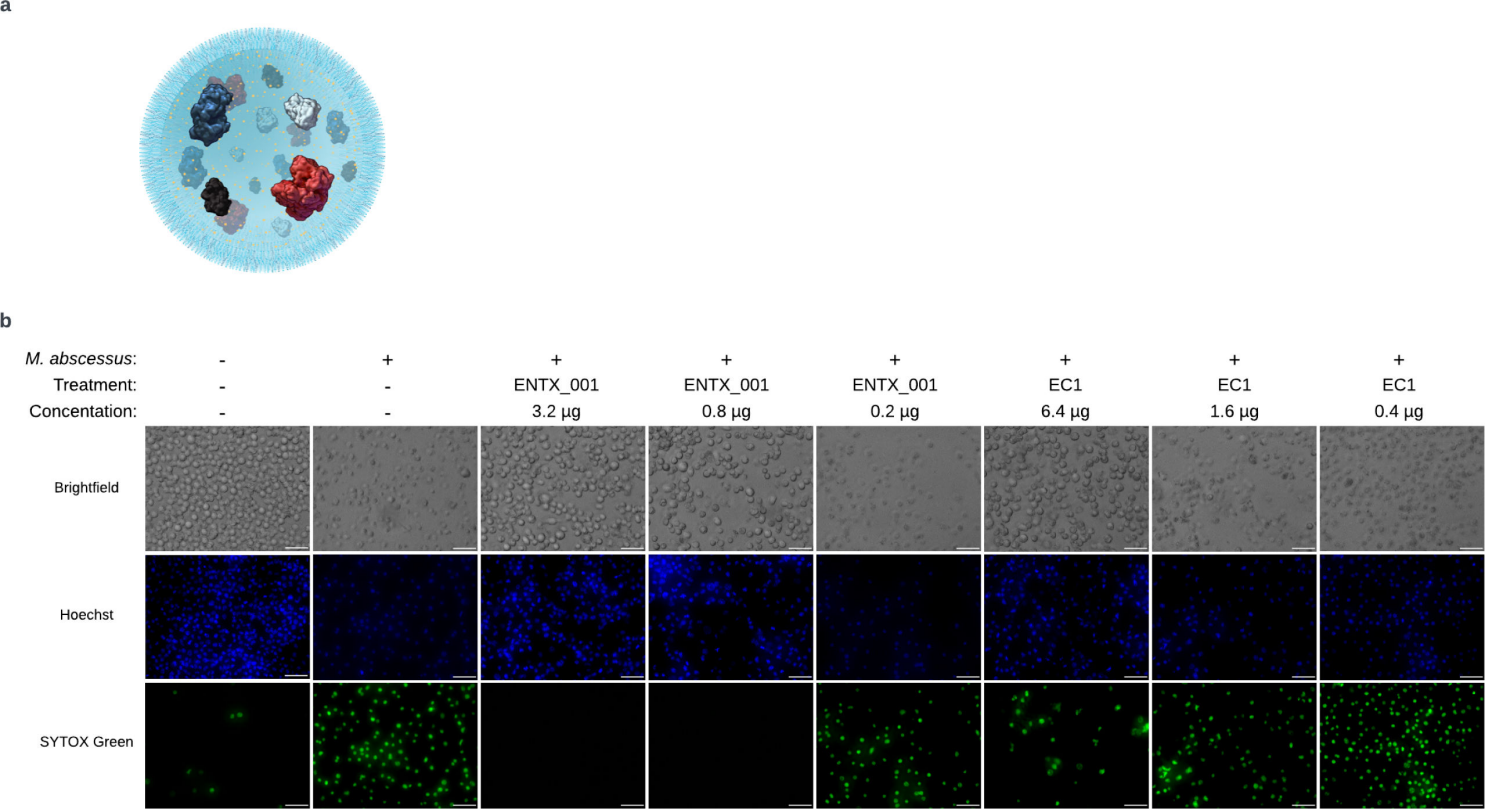
(a) Schematic diagram of ENTX_001 PPL. Lipids are light blue, small molecule excipients are yellow, the enzymes are PlyC in red (LysA representative), D29 LysB in black, *B. licheniformis* α-amylase in gray, and *C. glabrata* isoamylase in dark blue. (b) J774a.1 mouse macrophages or macrophages infected with *M. abscessus* str. 19977. IIM were treated with EC1 or ENTX_001 in a titration of protein concentrations indicated on the figure for 18 h and evaluated for necrosis by microscopy. After treatment, macrophages were stained with Hoechst (middle row, blue, total nuclei) and SYTOX Green (bottom row, green, necrotic cells) to measure necrosis. The scale bar is 50 µm.

Liposomal encapsulation of EC1 is the formulation we choose to provide access of the EC1 of enzymes to intracellular NTM thus creating our novel antibiotic ENTX_001, further defined as a poly-proteo-liposome (Fig. 6a). We encapsulated our proteins into liposomes via assembly in a NextGen microfluidic chip (Precision Nanosystems Instruments, Vancouver CA). The liposomes are built of physiological lipids including phosphatidyl serine that acts to stimulate macrophage engulfment via mimicking apoptotic cells (34). To evaluate encapsulation of ENTX_001, we demonstrated that enzymatic activity depended upon release of the ENTX_001 enzymes from the liposomal vehicle via low concentration non-ionic detergent (Triton X-100) liposomal lysis that does not kill NTM alone (Fig. S6).

We evaluated the necrotic state of J774A.1 mouse macrophages that were infected with *M. abscessus* at a multiplicity of infection (MOI) of 10:1 using fluorescence microscopy with stained nuclei (35). Hoechst Blue dye binds macrophage DNA in a membrane permeable fashion, whereas SYTOX Green dye binds DNA only when the cell membrane is compromised (36, 37). We then titrated either EC1 or ENTX_001 into IIMs prior to dual staining which led to the observation that the encapsulated PPL form of the drug demonstrated more potent protection of macrophages from necrosis than did the free enzyme cocktail, as observed by the prevention of SYTOX Green-labeled nuclei in a drug concentration-dependent manner (Fig. 6b).

**Fig 7:**
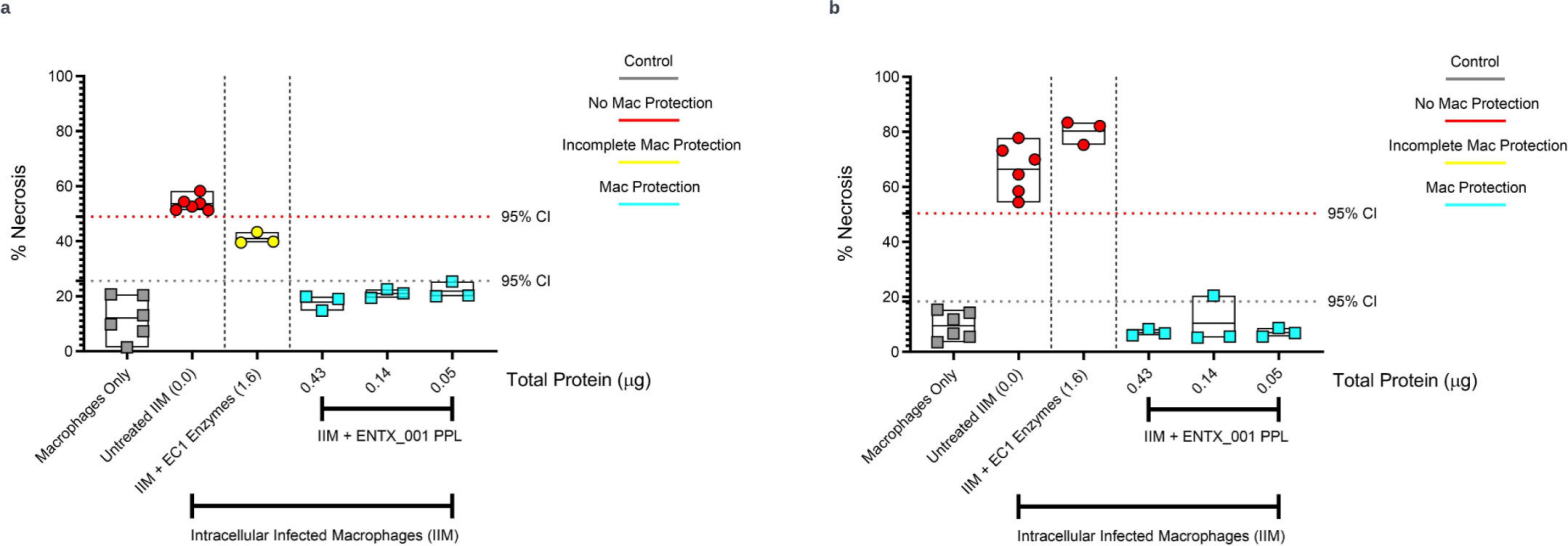
(a) Human macrophages (THP-1 cell line) were infected with *M. avium* str 2285R. IIM were treated with EC1 or ENTX_001 in a titration of protein concentrations for 24 h and evaluated for necrosis by flow cytometry resulting in three classifications ranging from no macrophage protection to incomplete and strong protection. The 95% confidence intervals are indicated with dotted lines for each class. (b) The figure is identical to panel a with the drug treatment time of 48 h.

To evaluate human macrophages for protection from mycobacteria-induced necrosis, we used the human macrophage cell line THP-1 for infection with *M. avium* 2285(R) at a multiplicity of infection of 10:1. Here, we show the ability of ENTX_001 to rescue macrophages from necrosis in the presence of infecting *M. avium* using flow cytometry (Fig. 7a and b). ENTX_001 protein contents and liposomal homogeneity via dynamic light scattering are shown in Fig. S7. Macrophage health was determined by running dual-stained macrophages through a flow cytometer followed by calculating the ratio of cells that are Hoechst positive versus those that are both Hoechst and SYTOX Green positive to quantify the *M. avium*-induced necrosis. In comparison to uninfected macrophages, ENTX_001 is capable of rescuing human macrophages from the necrotic fate of *M. avium* infection. We observed that the protection via the PPL formulation was far more potent and longer lasting than the enzyme cocktail alone. We also observed a similar pattern of macrophage protection in THP-1 cell lines when infected with M. *abscessus* strain 19977 (Mab) (Fig. S8). J774A.1 mouse macrophages also show similar phenotypes when infected with Mab (Fig. S9), demonstrating the ENTX_001 protection from infection-dependent macrophage necrosis is independent of bacterial and host species.

## DISCUSSION

In this study, we have shown the ability of the enzyme cocktail EC1 to kill NTM and *M. tuberculosis in vitro* for a diverse set of pathogenic mycobacteria and *M. abscessus*. The EC1 attacks mycobacteria at three layers in the envelope: the capsule, the mycomembrane, and the peptidoglycan layer through enzymatic degradation of the envelope. Our enzybiotic approach differs from small molecule antibiotics that act passively; instead, EC1 actively degrades the mycobacterial envelope regardless of microbial physiology and is insensitive to genome defense mechanisms such as restriction enzyme digestion and CRISPR systems. In addition, EC1 is not receptor limited like bacteriophage, thus expanding the applicable host range to both rapid-growing and slow-growing mycobacteria and closely related genera.

We have presented growth, microscopy, viability staining, and particle analysis data to demonstrate the ability of EC1 to shred mycobacteria into subcellular fragments that are separated from genomic DNA and prevent growth. The use of LysB-centered mycobacterial cocktail of enzybiotics was independently verified by a study in 2021 that used LysB, and α-amylase, against the envelope of M. *tuberculosis* (20); however, substantial activity required the addition of Tween 80, not required by EC1. A similar enzybiotic approach has also been used to attack the trehalose dimycolate, also known as cord factor, a lipid in the outer membrane of mycobacteria with another serine esterase trehalose dimycolate hydrolase further validating the mycobacterial envelope as a viable drug target (38, 39).

EC1 was inspired by and utilizes enzymes that have evolved in mycobacteriophages. The addition of capsule-degrading enzymes in support of attacking mycobacterial envelope from the outside in may further destabilize the envelope structure and provide greater access to the substrates for LysA and LysB. The bactericidal effect of our enzyme cocktail EC1 was further engineered to access targets inside of infected macrophages through targeted liposome encapsulation yielding ENTX_001. Our PPL formulation provides strong protection of macrophages, both mouse and human, from the necrotic death of infected macrophages *ex vivo*. This approach addresses the difficulty in drugging the intracellular compartment where mycobacteria grow and hide from chemical intervention and the adaptive immune system. By mimicking the lipid profile of apoptotic cells through the addition of phosphatidyl serine, we further target our liposomes to macrophages and their endocytic compartments.

This drug development program was designed to provide a way of killing mycobacteria in the host cells of infected patients where they dwell without the use of chemical antibiotics and the corresponding AMR. Given that treatment of mycobacterial infections involves prolonged therapy with regimens using multiple antibiotic cocktails, the observation of strong synergy between SoC antibiotics with our enzymatic cocktail suggests a reduction in the requisite concentration of chemical antibiotics when used in combination resulting in better patient tolerance and efficacy. ENTX_001 targets the intracellular seeds of infection raising the possibility of lowering the rate of infection reemergence post-therapy.

## MATERIALS AND METHODS

### *In silico* identification of LysA and LysB

We collected mycobacteriophage proteins from PhagesDB (371,279 proteins; accessed on 21 April 2021) using the provided API and custom scripts (40). Functional annotation in combination with protein homology search using Diamond BLASTp with a threshold of 90% sequence identity was used to identify 1,829 LysA proteins and 1,651 LysB proteins (41). Genetically similar proteins were clustered at 90% sequence identity using CD-HIT, then queried against the Conserved Domain Database using RPS-BLAST (42–44). Domains identified were clustered at 90% resulting in 27 domain archetypes for LysA. Similar search methods were used for LysB resulting in 14 domain archetypes. Additional thresholds are defined in supplemental methods.

### Source of capsule-degrading components of EC1

*Bacillus licheniformis* α-amylase was ordered from Sigma-Aldrich. We produced α-amylase from *Rhizomucor pusillus* in *Komagataella pastoris* using the standard workflow from ATUM. Isoamylase is derived from the fungi, *Candida glabrata*. The *Candida glabrata* isoamylase and *Rhizomucor pusillus* α-amylase were chosen based on the same desired features as LysA and LysB.

### Purification of protein components

LysA, LysB, and isoamylase genes were cloned into either a pET21a or pET24a + vector with a His6 tag. Isoamylase was cloned with a His10 tag. All plasmid vectors contained a lactose-inducible T7 promoter by GenScript. Plasmids were transformed into *Escherichia coli* BL21 and grown in Terrific broth with 100 µg/mL carbenicillin or 50 µg/mL kanamycin and induced at 16°C with 0.1 mM isopropyl β-d-1-thiogalactopyranoside (IPTG) or an auto induction method using 0.1% glucose and 0.4% lactose. Induction media was also supplemented with 25 mM ammonium sulfate, 50 mM potassium dihydrogen phosphate, 50 mM disodium phosphate, 0.5% glycerol, and 1 mM magnesium sulfate. After induction, cells were harvested by centrifugation, resuspended in lysis buffer, then homogenized for 3 min using a Fisher homogenizer at 15 K rpm. One microliter Benzonase (2.5 kU stock) per 100 mL lysate and 1 µL lysozyme (5 mg/mL stock) per 20 mL lysate were mixed with lysate for 1 h at room temperature and lysed by two passes over an LM20 microfluidizer at 15 K psi. The soluble fraction was harvested by centrifugation at 18,500 × *g* for 30 min then purified using Ni Sepharose 6 FF IMAC resin equilibrated with lysis buffer (Cytiva, Marlborough, MA). Column-bound protein was then eluted in lysis buffer containing 300 mM imidazole and buffer exchanged by tangential flow filtration (TFF) to remove imidazole. Proteins were stored at −80C in storage buffer (SB) containing 50 mM glycine, 200 mM NaCl, 0.5 mM MgCl_2_, and 20% glycerol at pH 8.3.

*Rhizomucor pusillus* α-amylase produced in *Komagataella pastoris* was purified by ion-exchange chromatography. Harvested culture broth was buffer exchanged by TFF into 50 mM glycine buffer, pH 8.5 containing 20 mM NaCl. The sample was applied to a Q Sepharose High Performance resin and eluted using an increasing salt gradient. Fractions collected were analyzed by SDS-PAGE and pooled based on homogeneity. Proteins were stored at −80C in SB.

### Thermal stability characterization of EC1 enzymes

The Uncle instrument from Unchained Labs was used to evaluate biophysical properties of target enzymes. Purified proteins were subjected to a thermal ramp from 25°C to 95°C at a rate of 0.5°C per minute. The intrinsic fluorescence profiles were used to determine *T*_*m*_ and static light scattering signals were used to determine the *T*_agg_.

### Liposome encapsulation of EC1

In addition, 3.2 mg/mL of each LysA, LysB, isoamylase, and α-amylase was mixed to achieve a final total protein concentration of 12.8 mg/mL total protein in a buffer containing 50 mM glycine, pH 8.50, 200 mM NaCl, 0.5 mM MgCl_2_, 0.33 mM sodium citrate, 7.5 mM CaCl_2_, and 10% glycerol. Buffer components were purchased through Fisher Scientific. 1,2-Dioleoyl-sn-glycero-3-phosphocholine (33.5%), 1,2-dioleoyl-sn-glycero-3-phosphoethanolamine (33.5%), 1,2-dioleoyl-sn-glycero-3-phospho-L-serine (DOPS; 13%), and cholesterol (20%) were purchased through Avanti Polar Lipids and mixed in 100% ethanol to a final concentration of 3.3 mg/mL. The protein solution and lipid mix were assembled into liposomes using Precision NanoSystems NanoAssemblr Ignite (Vancouver, BC, Canada) and the payloaded liposomes were purified away from unencapsulated proteins and other material <750 kDa using TFF. Liposome diameter and polydispersity index were analyzed with the Malvern Zetasizer Ultra (Malvern Panalytical, Malvern, UK), and final protein compositions were evaluated with SDS-PAGE stain-free gels (Bio-Rad).

### Mycobacterial culture

Mycobacteria were cultured according to standard practice and fully described in the supplemental methods.

### Drug susceptibility testing (DST) and CFU enumeration

*M. abscessus* strain ECL14 was harvested following overnight growth in 7H9 OADC at 3,500 rpm for 10 min, washed (1×), and resuspended in reaction buffer (RB): prepared fresh by diluting from a (5×) stock (pH 8.50) comprised of 500 mM L-arginine hydrochloride, 100 mM calcium dichloride dihydrate, 250 mM glycine, 2.5 mM magnesium chloride hexahydrate, 5 mM sodium citrate dihydrate, 1M sodium chloride, and 15% (vol/vol) in milli-Q H_2_O, passed through 0.20 µm membrane filter, adjusted to an optical density at 600 nm (OD_600_) of 0.025 (i.e., 3.75 × 10^7^ CFU mL^−1^), and sonicated using the Pixul sonicator with the pulse set to 25.00, pulse repetition frequency equal to 1.0, process time equal to 15 min, and burst rate set to 1 to create a single cell resuspension of mycobacteria to disaggregate mycobacteria. Sonication conditions were demonstrated to be non-lethal to mycobacteria and validated in comparison to needle disaggregation with and without enzymatic treatment (data not shown). Frozen aliquots of lysin A (A), D29 lysin B (B), isoamylase (I), and α-amylase (α) were thawed then put on ice until diluted or added to reaction wells. Proteins were diluted to working stocks in storage buffer, pH 8.30. Thirty-two microliters of (1×) RB was aliquoted into sample reaction wells in 96-well round bottom non-tissue culture-treated assay plates, followed by 8 µL (i.e., ~2.5 × 10^5^ CFU) mycobacteria and 10 µL of protein(s), as necessary. Ten microliters of RB was added to drug free (DF) control wells in plates. Fifty microliters DST reactions was mixed by gently pipetting up and down (5–10×) with a P20 multichannel micropipette, then sealed with “Breath Easy” breathable seals to avoid dehydration and incubated overnight at 37°C, 5% CO_2_, stationary, in a tissue culture incubator.

### *In vitro* grow-out of serial dilutions after EC1 exposure

*M. abscessus* grown to log phase (OD_600_ = 0.10) in 7H9 broth was briefly collected for 5 min at 5,000 rpm at 25°C and resuspended in (1×) RB, 100 mM L-arginine, 20 mM calcium chloride, 0, 10, 20, 30, 40, and 50 mM glycine (pH 8.50) to achieve pH range for enzymatic reactions, 0.5 mM magnesium chloride, 1 mM sodium citrate, 200 mM sodium chloride, and 15% (vol/vol) glycerol. The OD_600_ of the bacterial resuspension was adjusted to OD_600_ of 0.1 and sonicated. The OD was adjusted to 0.1 (~1.5E^8^ cells per milliliter). Five microliters of single cell resuspension in RB was used to inoculate a 50 µL reaction (final volume), with 3.2 µg of free EC1 proteins in equal mass ratios with 1E+05 CFU of Mab in Biolog plates, and observed with kinetic measurements of Omnilog Units at 37°C in Omnilog (Biolog, Inc.) (45), sealed with “Breath Easy” seals (Sigma-Aldrich) for 5 days.

### Nontuberculosis mycobacteria MIC

MIC assays were carried out using the microbroth dilution method per CLSI guidelines (46). NTM strains were grown in 7H9 media (Fisher) plus 10% albumin dextrose catalase (ADC) supplement (Fisher) to mid-log phase and diluted to an OD_600_ of 0.1 in cation-adjusted Mueller-Hinton broth, CAMHB (Fisher). Slow-growing NTM strains were diluted in CAMHB + 10% OADC supplement (Fisher). Cultures were diluted again 1:200 in CAMHB to achieve a 5E^5^ CFU/mL inoculum concentration. Drug or protein cocktail was diluted 1:2 in CAMHB in a 96-well plate and, once mixed 1:2 with the inoculum, will yield a concentration range of 64–0.25 µg/mL for *M. tuberculosis* strains SoC drugs, 32–0.125 µg/mL for NTM strains, and 12.8–0.025 µg for protein cocktail for all strains in a total volume of 100 µL per well. MIC plates were inoculated in triplicate for 72 h at 37°C for rapid-growing NTM strains and 7 days at 37°C for slow-growing NTM strains. MIC is the lowest concentration to show no growth in a well compared to untreated growth control wells from visual inspection.

### FICI determination

FICI was determined by using a checkerboard MIC assay. Protein cocktail dilutions were made in a 96-well plate left to right at a 4× final concentration, 25 µL volume. Drug dilutions were made in separate 96-well plates top to bottom at a 4× final concentration, 50 µL final volume. Twenty-five microliters of drug dilutions was added to the protein cocktail dilutions. A final 50 µL volume of inoculum was added to each protein cocktail/drug mixture, and plates were incubated as noted in the MIC assay section. MIC of the combo treatment was recorded as the lowest concentration of each drug to inhibit growth compared to untreated growth control. The FICI was calculated using the following equation:


$$FICI=\frac{MIC\;combo}{MIC\;drug}+\frac{MIC\;combo}{MIC\;protein\;cocktail}$$

A FICI score of <0.5 denotes synergism, 0.5–1 denotes additivity, 1–4 denotes indifference, and >4 denotes antagonism.

### Imaging and image analysis of EC1 reactions *in vitro*

After enzymatic treatment of 1E^5^ NTM cells, 50 µL of the reaction was mixed with 50 µL of a stain mix containing 10 mM of SYTOX Green Nucleic Acid Stain (SG) (Invitrogen) and 0.25 mM FM4-64 (FM) (Invitrogen) in PBS in a black 96-well plate (Corning CLS3603). The plate was centrifuged for 2 min at 3,000 rpm and imaged with a Keyence BZ-X810 Fluorescence Microscope at 40× magnification. Unprocessed images were overlaid and resized using Photoshop 2022.

### Flow cytometry of EC1 reactions

For *in vitro* testing, 1E+07 CFU bacteria were collected from pooled samples at 5,000 rpm for 10 min at 25°C, and resuspended in Dulbecco’s phosphate buffered saline (DPBS), supplemented with 10 mM of SYTOX Green Nucleic Acid Stain (SG) (Invitrogen) and 0.25 mM FM4-64FX (FM) (Invitrogen) and incubated at 37°C for 30 min. Stained samples were spun at 5,000 rpm for 10 min at 25°C and resuspended in DPBS, with 2% paraformaldehyde. Flow cytometry was performed on a CellStream flow cytometer (Cytek Biosciences) in small particle detection mode, with flow rate set to 3.66 µL a min and no thresholds, to separate bacteria from debris. Lasers with excitation wavelengths at 450 nm, 488 nm, and 785 nm were used for detection; SG fluorescence was detected in 528/46 nm band pass filter, while FM fluorescence was detected in 702/87 nm band pass filter. Unstained and single-stained DF controls were run on CellStream and compensation was applied prior to running dual-stained samples. Unstained DF controls were run on the CellStream to adjust fluorescence intensity of 488 nm laser, such that unstained events appeared within the first order of the log scale of fluorescence. Forward-scattered (FSC) versus side-scattered (SSC) light were applied to separate 5,000 particles counted for every sample into populations of larger aggregates, debris, and single bacteria. SG versus FM fluorescence intensity was assessed with bivariate plots of each gated population.

### Macrophage protection assays

For *ex vivo* testing, 5E+04 (J774A.1 and THP-1) cells resuspended in fresh complete media (cDMEM or cRPMI) were seeded in wells of a 96-well tissue culture-treated flat bottom plate(s) and incubated at 37°C for 16–18 h. For THP-1 human cells, 40 nM of phorbol 12-myristate 13-acetate (ThermoFisher Scientific) was also added to cRPMI. The next day, spent media was replaced with fresh cRPMI or cDMEM and macrophages were infected with 5E+05 CFU (10:1 MOI) of Mab grown up to an OD_600_ of 0.1 in 7H9, washed (1×) and resuspended in DPBS, and sonicated for 3 h at 37°C. Extracellular bacteria were removed by aspiration, and subsequent treatment for 1 h at 37°C in fresh cDMEM or cRPMI, supplemented with 250 µg per mL of amikacin (47, 48). Cells were then washed with DPBS, replenished with fresh complete media, containing 50 µg per mL of amikacin, treated, and incubated for 16–20 h at 37°C. The following day, for flow cytometry, wells were washed with DPBS, replenished with fresh cDMEM or cRPMI, supplemented with 8 µM Hoechst 33342 Solution (Invitrogen) and 1 µM of SG (Invitrogen), and incubated in the dark for 30 min at 37°C. Staining media was removed, cells were washed with DPBS, 125 µL of StemPro Accutase (ThermoFisher Scientific) was added to wells, and plates were incubated at 37°C for 30 min. An equal amount of eBioscience Flow Cytometry buffer (Invitrogen), with 4% paraformaldehyde (Sigma-Aldrich), and fixed stained cells were analyzed on a CellStream (Cytek Biosciences), using a flow rate of 14.66 µL/min, with thresholds of FSC and SSC set to 1,000 to separate cells from debris. Lasers with excitation wavelengths at 405 nm, 450 nm, 488 nm, and 785 nm were used for detection; Hoechst fluorescence was detected in the 456/51 band pass filter, while SG fluorescence was detected as described before. Unstained and single-stained drug-free controls were run on CellStream and compensation was applied prior to running dual-stained samples. Unstained and single-stained control samples were run on the CellStream to adjust fluorescence intensities of 405 nm and 488 nm lasers, such that unstained events appeared within the first order of the log scale fluorescence. FSC versus SSC plots were initially used to separate 1,500 particles counted per sample into populations of cells and debris. Percent viability was assessed by evaluating SG/Hoechst-positive sub-populations compared to untreated and infected cells. For microscopy analysis, the following day after treatment, the media was removed and replaced with 8 µM Hoechst and 1 µM SYTOX Green in cDMEM. The cells were stained for 30 min, washed twice with DPBS, then imaged on a Keyence BZ-X800 with a 40× objective. Unprocessed images were overlaid and resized using Photoshop 2022.

## SUPPLEMENTAL MATERIAL

Supplemental figures spectrum.03534-23-s0001.pdf Fig. S1 to S9.

Fig. S10 spectrum.03534-23-s0002.docx FICI scores.

Supplemental text spectrum.03534-23-s0003.docx Supplemental methods.

Supplemental table spectrum.03534-23-s0004.xlsx Table S1.

ASM does not own the copyrights to Supplemental Material that may be linked to, or accessed through, an article. The authors have granted ASM a non-exclusive, world-wide license to publish the Supplemental Material files. Please contact the corresponding author directly for reuse.
