# Supplementary material for: Targeting intracellular nontuberculous mycobacteria and M. tuberculosis with a bactericidal enzymatic cocktail
Source: Microbiol Spectr. 2024 Mar 27;12(5):e03534-23. doi: 10.1128/spectrum.03534-23 (PMC11064574; doi:10.1128/spectrum.03534-23)
Supplement: Supplemental figures — Fig. S1 to S9. [file spectrum.03534-23-s0001.pdf]

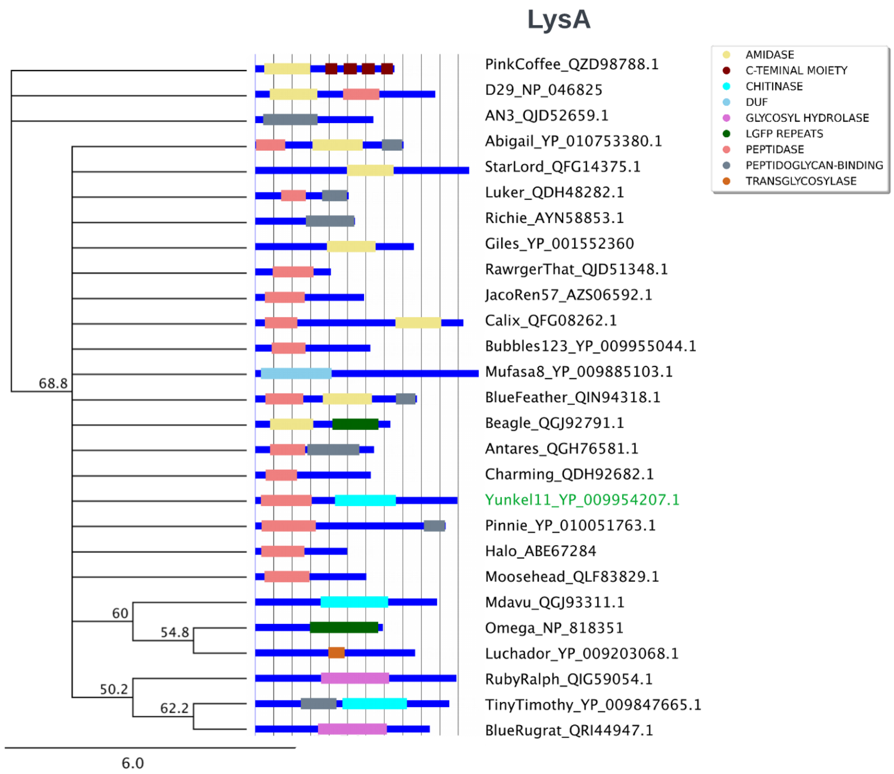

Supplemental Figure 1: LysA phylogenetic tree and archetypes comprised of identified domains within the protein members of each phylogenetic clade. Grey vertical lines represent 50 amino acids. Protein Names and accession numbers are displayed.

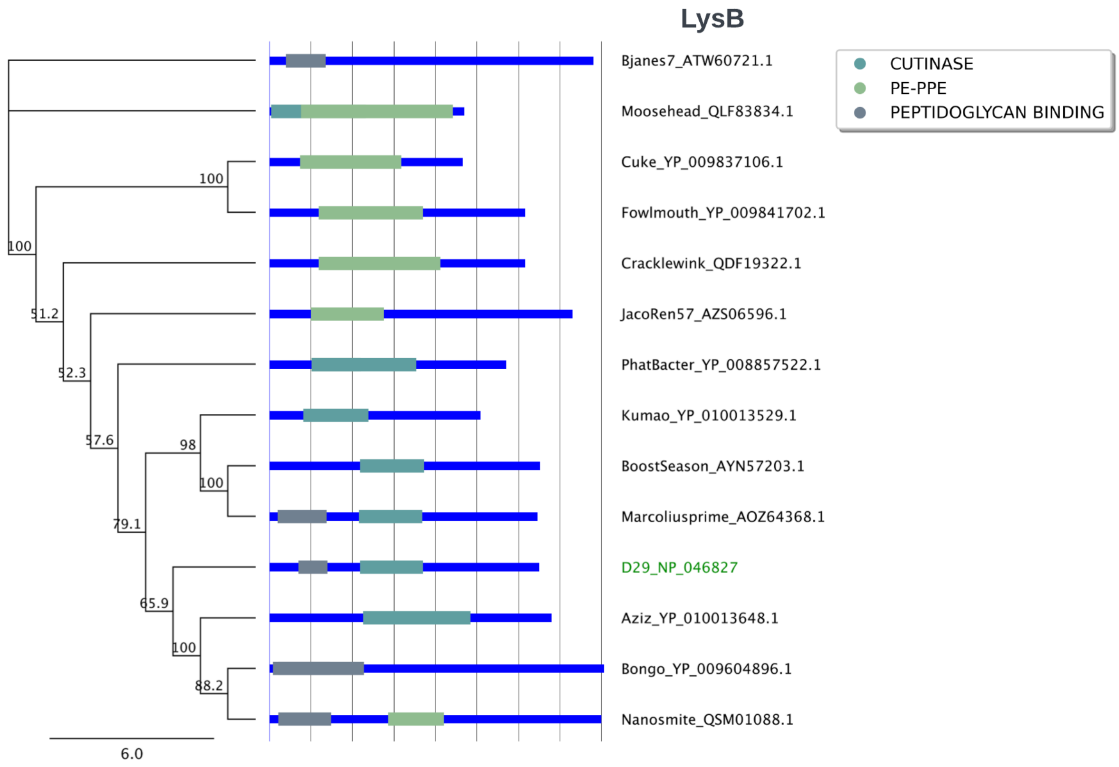

Supplemental Figure 2: LysB phylogenetic tree and archetypes comprised of identified domains within the protein members of each phylogenetic clade. Grey vertical lines represent 50 amino acids. Protein Names and accession numbers are displayed.

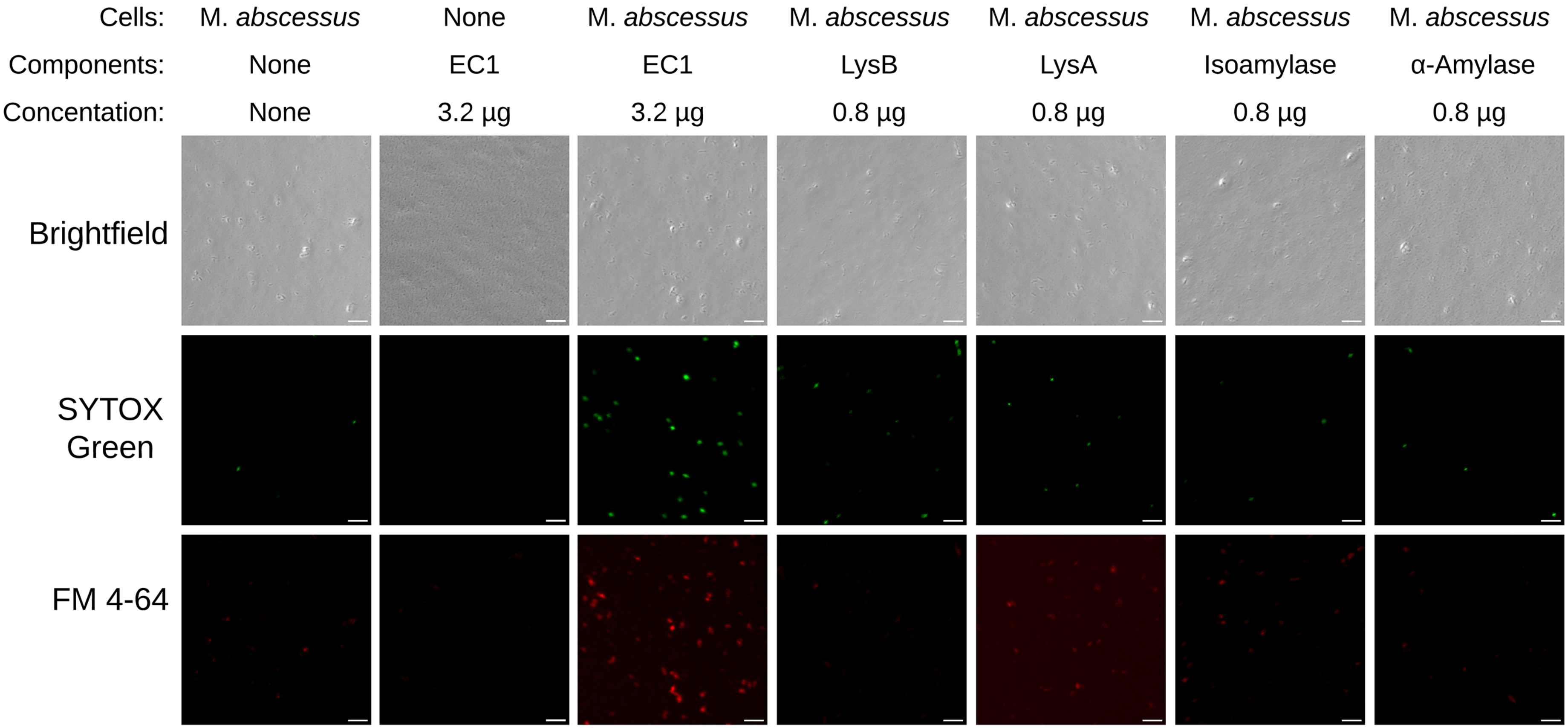

Supplementary Figure 3: Brightfield and fluorescence microscopy images of in vitro EC1 reactions with M. abscessus. The top panel contains bright field images; whereas the middle panel displays only SYTOX Green signal that binds nucleic acids. The bottom panel displays only the FM 4-64 signal, that binds to the outer leaflet of the plasma membrane. The scale bar is 10 µm.

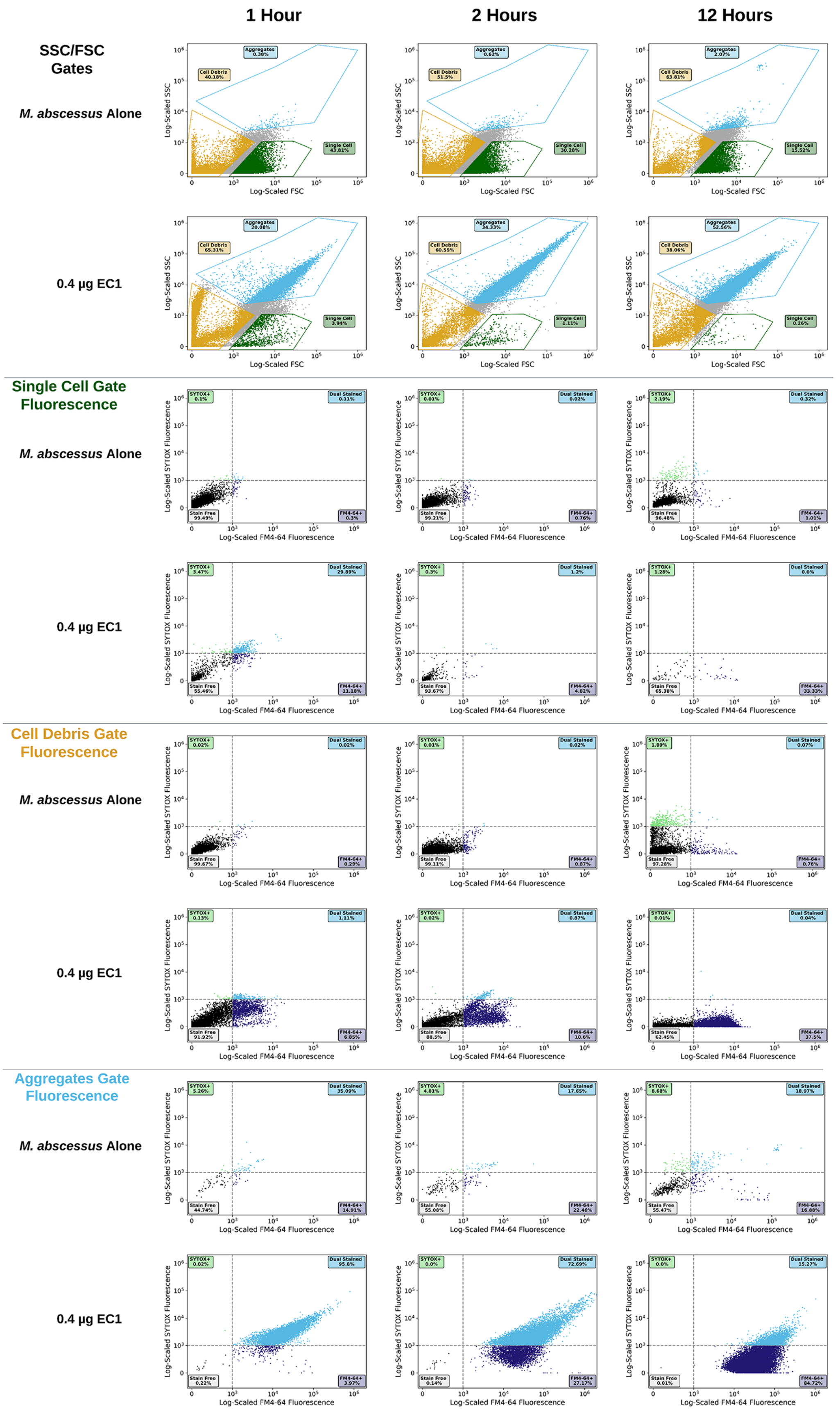

Supplemental Figure 4: Characterization via flow cytometry of mycobacterial populations of single cells, small particles, and aggregates at t=1, 2, and 12 hours after treatment with EC1 in vitro. Cells and the resulting enzymatic products were stained with SYTOX Green Nucleic Acid Stain and FM4-64 mycobacterial lipid membrane stain. Mycobacterial populations (i.e., single cellular debris, and aggregates were detected using FSC & SSC to reveal size and features in the top two rows. Graphs of particle size/ feature were used to establish gates for fluorescent analysis plots of treated and untreated cell populations, unstained or stained with FM4-64 and/or SYTOX as percentages ratios. For rows 3-8, the x- and y-axes represent increasing FM4-64 and SYTOX fluorescence intensity, respectively, in logarithmic scale and range incrementally from 103-to-106.

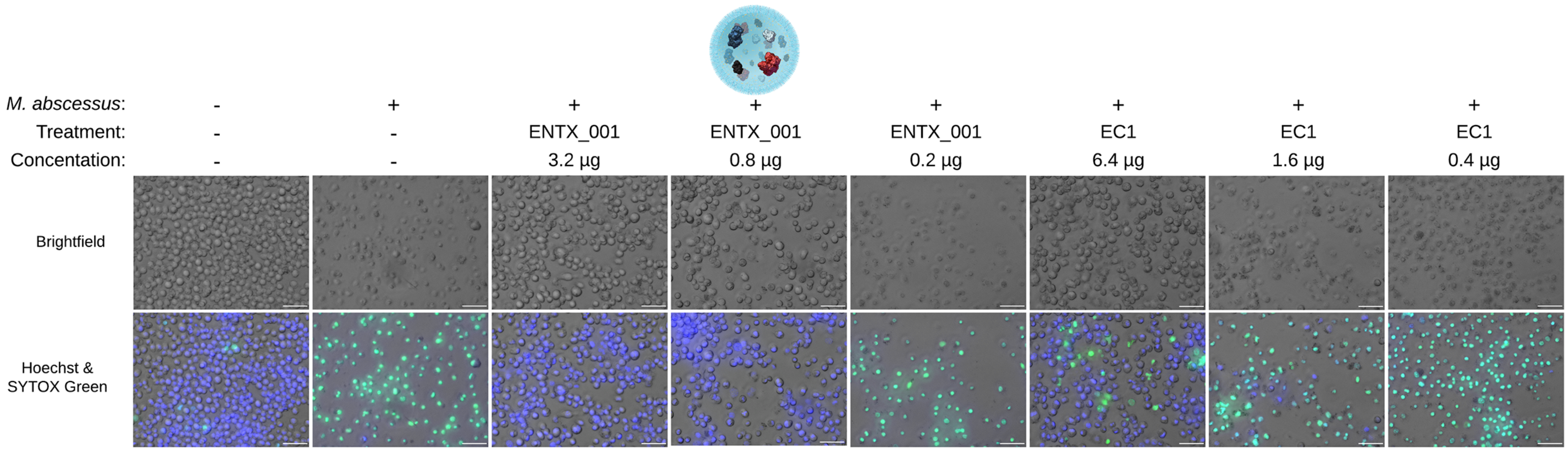

Supplemental Figure 5: J774a.1 mouse macrophages or macrophages infected with M. abscessus str. 19977 Intracellular Infected Macrophages (IIM) were treated with EC1 or ENTX_001 in a titration of protein concentrations indicated on the figure for 18 hours and evaluated for necrosis by microscopy. After treatment, macrophages were stained with Hoechst (Blue, total nuclei) and SYTOX Green (Green, necrotic cells) and presented as a merged view if green and blue signal to measure necrosis (bottom row). Images in the top row are brightfield images. The scale bar is 50 µm. This figure supports Figure 6.

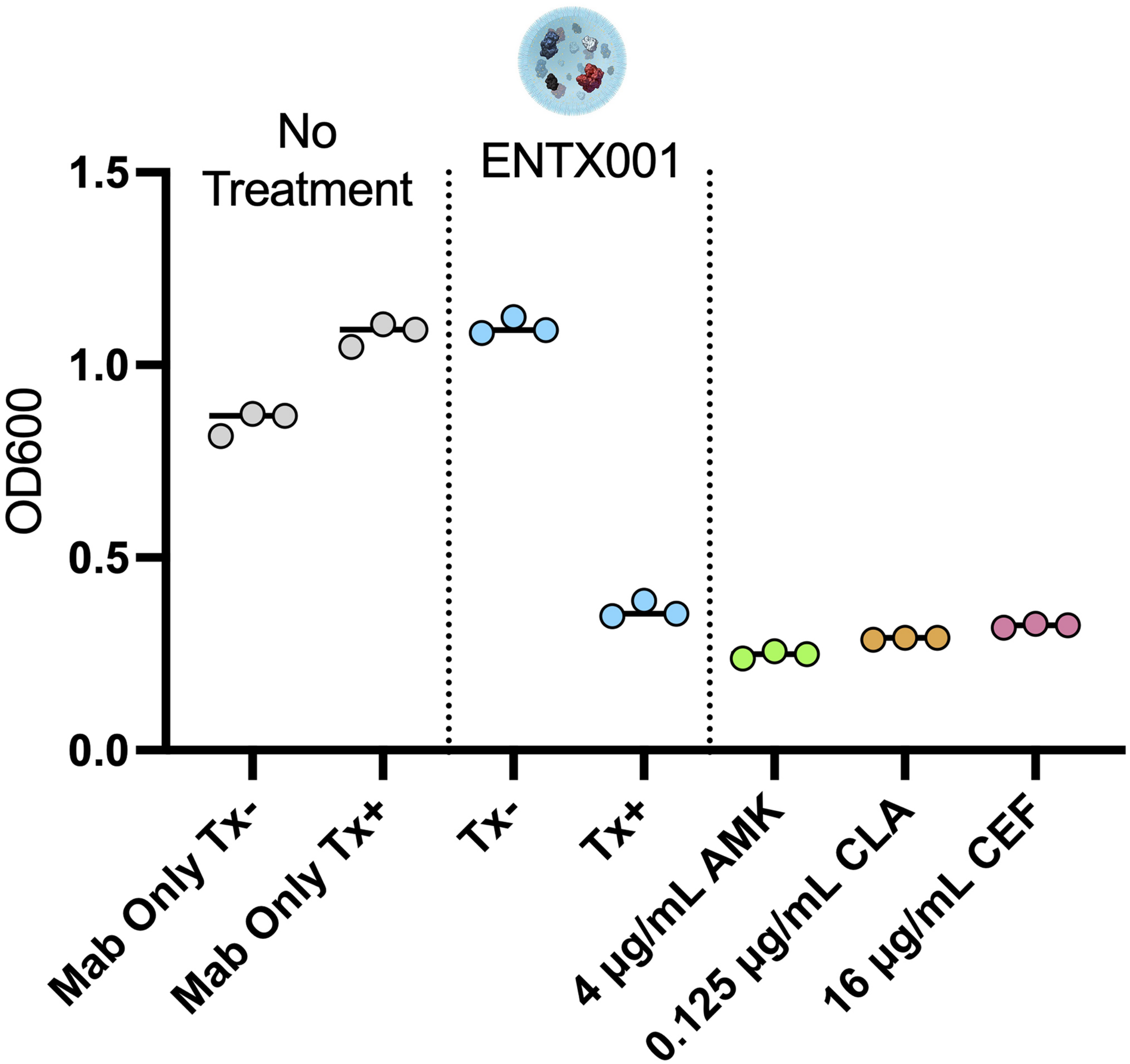

Supplemental Figure 6: Liposomal lysis using sublethal concentration of Triton X-100 shows the enzymes remain active after lipid encapsulation, but inactive until released from the liposome. The effects on growth were measured with OD600 readings and compared to above MIC levels of Amikacin (AMI), Clarithromycin (CLA), and Cefoxitin (CEF).

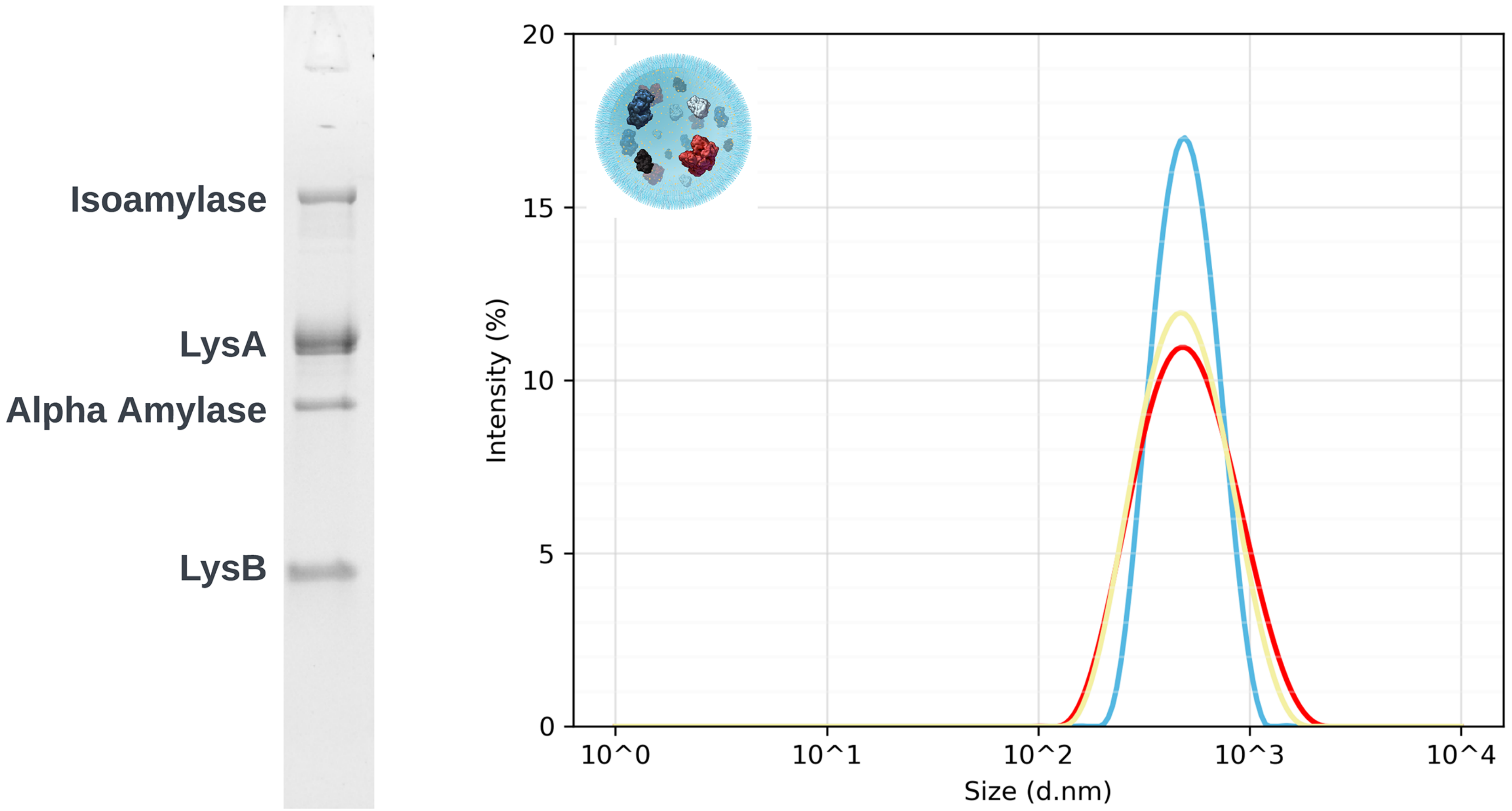

Supplemental Figure 7: ENTX_001-PPL features of protein composition are displayed by SDS-PAGE and Dynamic Light Scattering (DLS). This figure supports Figure 7 containing an alternative α-amylase derived from Rhizomucor pusilus. All other protein components are the same. ENTX_001-PPL features of protein composition are displayed by SDS-PAGE and Dynamic Light Scattering (DLS). The diameter of the liposome Z-Average 442 +/- 8 nm, Peak is 541 +/- 25 nm, with the Poly Dispersity Index PDI 0.23 +/- 0.01.

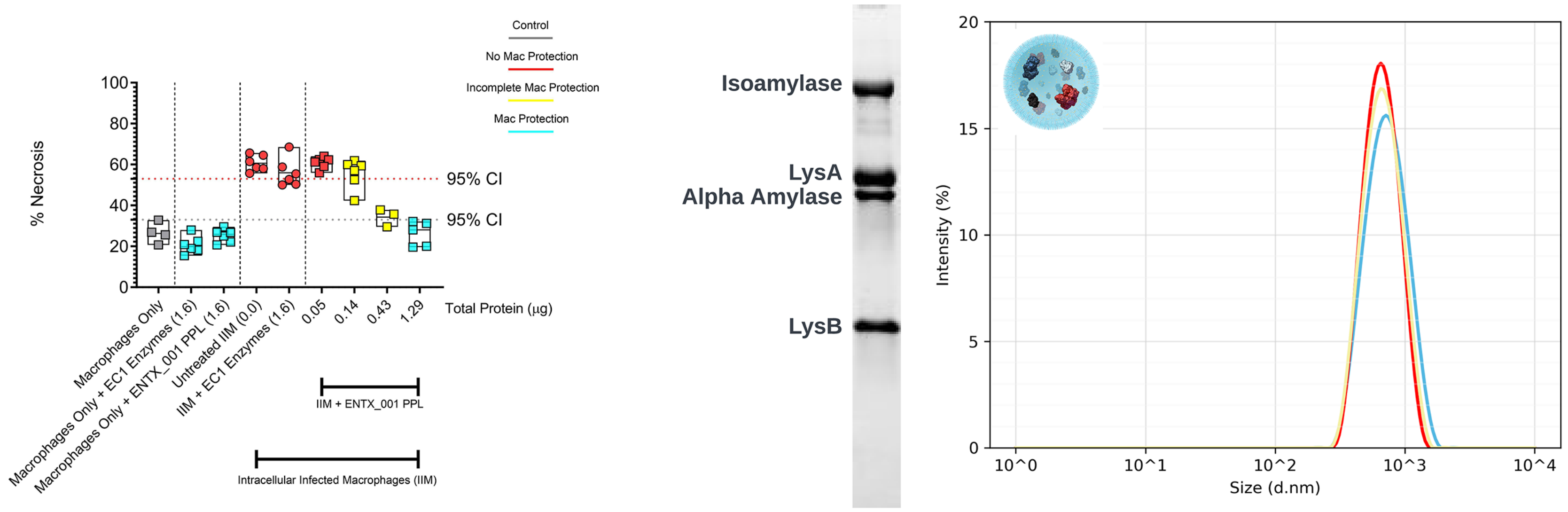

Supplemental Figure 8: A titration of EC1 versus ENTX_001 in human macrophages (THP1) infected with M. abscessus evaluated by flow cytometry for the presence of necrotic nuclei/ total nuclei with SYTOX Green/Hoechst. ENTX_001-PPL features of protein composition and purity are displayed by SDS-PAGE and DLS. The diameter of the liposome Z-Average 547 +/- 30, PDI 0.267 +/- 0.030, Peak Mean 715 +/- 38.

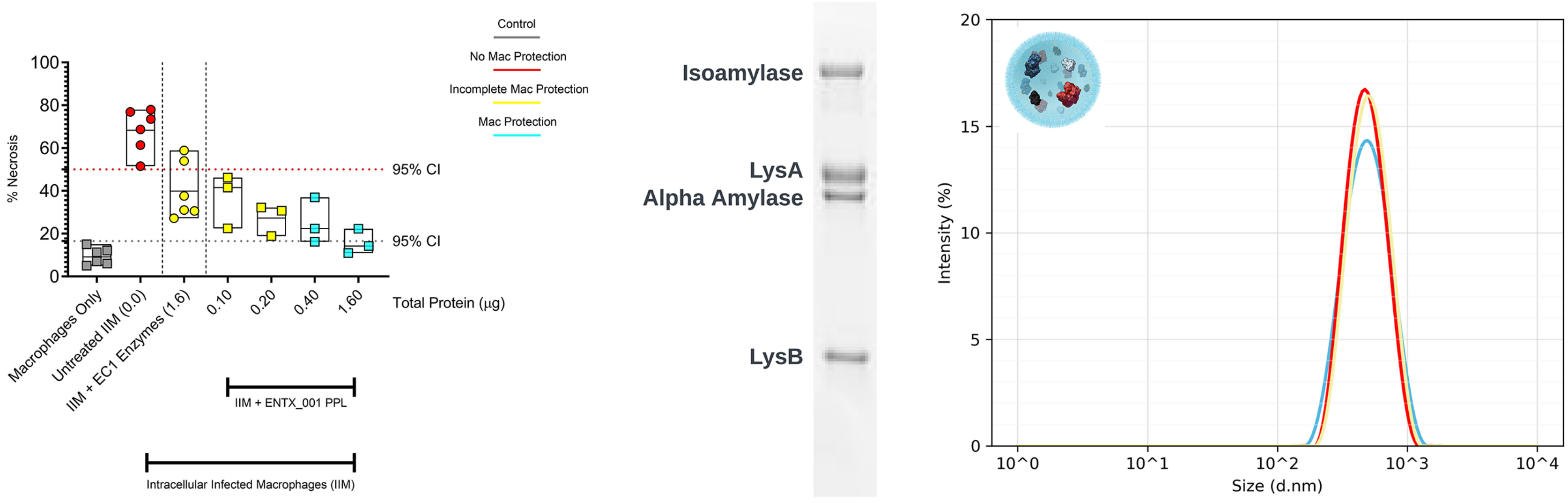

Supplemental Figure 9: A titration of EC1 versus ENTX_001 in mouse macrophages (J774A.1) infected with *M. abscessus* evaluated by flow cytometry after 24 hours of treatment for the presence of necrotic nuclei/ total nuclei with SYTOX Green/Hoechst. ENTX_001-PPL features of protein composition are displayed by SDS-PAGE and DLS. The diameter is Z-Average 417 +/- 2.1, Peak Mean 519 +/- 14.7, PDI 0.23 +/- 0.01.
