# Supplementary material for: Targeting intracellular nontuberculous mycobacteria and M. tuberculosis with a bactericidal enzymatic cocktail
Source: Microbiol Spectr. 2024 Mar 27;12(5):e03534-23. doi: 10.1128/spectrum.03534-23 (PMC11064574; doi:10.1128/spectrum.03534-23)
Supplement: Fig. S10 — FICI scores. [file spectrum.03534-23-s0002.docx]

Supplemental Figure 10: FICI scores representing combinations of EC1 and SoC drugs on rapid and slow growing mycobacteria as well as *M. tuberculosis. M. abscessu*s 19977 and 103, *M. avium* Mac 101 and *M. avium* 2285R, *M. intracellulare* ECL 55, *M. avium* ECL 94, *M. tuberculosis* H37Rv and Tn587. The drugs used were EC1, Clarithromycin (CLA), Moxifloxacin (MOX), Cefoxitin (FOX), Imipenem (IMI), Amikacin (AMI), Isoniazid (INH), and Rifampicin (RIF). The table is color coded in 3 classes: Synergistic (green) and additive (blue).
