# Supplementary material for: Targeting intracellular nontuberculous mycobacteria and M. tuberculosis with a bactericidal enzymatic cocktail
Source: Microbiol Spectr. 2024 Mar 27;12(5):e03534-23. doi: 10.1128/spectrum.03534-23 (PMC11064574; doi:10.1128/spectrum.03534-23)
Supplement: Supplemental text — Supplemental methods. [file spectrum.03534-23-s0003.docx]

**Supplemental Methods**

Bioinformatic Selection Criteria

Genetically similar proteins were clustered at 90% sequence identity using CD-HIT to create 224 unique amino acid clusters (1). We queried a representative of the 224 clusters using RPS-BLAST against the full Conserved Domain Database (CDD) (2,3). An e-value threshold below 10^-5 was used to filter domain hits. If multiple domains were identified in the same region, the domain with the highest confidence score was retained. Identified domain sequences were extracted from the protein and concatenated. These domain sequences were again clustered at 90% sequence identity using CD-HIT to identify domain archetypes. These domain archetypes were cloned into a modified pET21a or pET24+ vector and further tested for expression.

Keyence Microscope Imaging Settings

Images were captured using a Keyence All-in-One Fluorescence Microscope BZ-X800 with Keyence Analyzer (v1.1.2.4). Hoechst and FM4-64 channel lasers were set to full strength (100) and minimal contrast (0). The laser strength of the SYTOX channel was reduced to 45 and contrast adjustment was set to full (100). A haze reduction function was applied with a radius of 10, a rate of 1.8, and a strength of 0.70. Scale bars were added to the raw images generated using ImageJ (v1.53t) with Java (v1.8.0_345). The scale for images with 60x magnification is 0.126 µm/pixel and 0.189 µm/pixel for 40x magnification. Unprocessed images with added scale bars were aligned and resized in Adobe Photoshop 2022.

Culturing Mycobacteria

Mycobacteria were grown at 37°C with shaking at 225 rpm in Middlebrook 7H9 liquid broth (Becton Dickinson), supplemented with 10% (vol/vol) albumin-dextrose-catalase (Becton Dickinson) enrichment, 0.50% (vol/vol) glycerol and 0.05% (vol/vol) Tween-80 (Sigma-Aldrich). Mab was propagated as a smooth colony morphotype at 37°C on Middlebrook 7H10 agar, supplemented with 10% (vol/vol) albumin-dextrose-catalase enrichment. J774A.1 cells (ATCC) were cultured in complete (c)DMEM (Gibco), supplemented with 10% (vol/vol) FBS, 100 U per mL penicillin, and 10 mg per mL streptomycin, and passaged every 2-3 days. THP-1 cells (ATCC) were cultured in complete (c) RPMI, supplemented with 10% (vol/vol) FBS, 55 µM 2-Mercaptoethanol, 100 U per mL penicillin, and 10 mg per mL streptomycin, and passaged every 3-4 d.

Genome Library Preparation and Genomic Sequencing

Single colonies (i.e., N=3) of ECL55 and ECL94 strs. grown up on 7H10 Middlebrook agar at 37^o^C for 7 d were picked, using sterile loops, and transferred to 1.50 mL Eppendorf tubes, with 1.00 mL of PBS. After spinning down at 3,000 rpm for 10 min at 25^o^C and removing PBS, tubes were heated to 80^o^C for 60 min. CFU were resuspended in 6.00 mL of 50 mM Tris-HCl, 25 mM EDTA, and 5.00% (v/v) monosodium glutamate, pH 7.40, transferred to 50 mL conical tubes, with glass beads (i.e., 5 mm diameter), and vortexed for 5 min. 400.00 µL of lysozyme (i.e., 50 mg mL^-1^) and 10.00 µL of RNase A (i.e., (10 mg mL^-1^) were added, and tubes and left at 37.00^o^C for 2 h, with gentle agitation. Afterwards, 600.00 mL of 100 mM Tris-HCl, 50 mM EDTA, and 5.00% (v/v) SDS, and 150.00 µL of proteinase K (i.e., 10.00 mg mL^-1^) was added, mixed by inversion, and left at 45^o^C for 16 h. The next day, 5.00 mL of Phenol:Chloroform:Isoamyl Alcohol (25:24:1) was added to tubes, mixed by inversion over 30 min, and centrifuged at 3,000 *X g* for 20 min at 25^o^C. The upper aqueous phase from each tube was transferred to 15 mL conical tubes, containing 0.10x vol. of 3 M sodium acetate, pH 5.50. An equal amount of isopropanol was then added to each tube, mixed by inversion, incubated overnight at -20.00^o^C, centrifuged at 3000 *X g* for 30 min at 25^o^C, washed in 70.00% (v/v) ethanol, air-dried, and dissolved distilled water.

Samples were sequenced with 2x150bp short reads using an Illumina MiSeq (Azenta Life Sciences). Reads were assembled using unicycler version 0.5.0 in standard operating mode for a short read only assembly. Blast was used against the resulting contigs with a *Mycobacterium avium* complex only database to validate that the genomes are likely *Mycobacterium intracellulare*.

Genome Assembly and Annotation

Samples were sequenced with 2x150bp short reads using an Illumina MiSeq (Azenta Life Sciences). Reads were assembled using unicycler version 0.5.0 in standard operating mode for a short read only assembly (4). Speciation was initially performed using NCBI’s ANI. Genomes were then annotated using NCBI’s PGAP with species chosen based on a mix of high ANI and coverage. Kraken2 was also ran on short reads as a secondary speciation (5). Species for an orthology analysis were then chosen based on being a top hit from kraken2 or ANI, as well as having a complete genome in NCBI. M. tuberculosis was also used to represent an anchor for the resulting speciation tree. Orthology was then generated for these species using orthofinder, and the resulting orthology was visualized using iTOL.

Live/Dead Off Target Assay

Bacterial cultures grown to log phase were centrifuged for 5 min at 5000 RPM then resuspended in reaction buffer (100 mM L-arginine, 20 mM calcium chloride, 0, 10, 20, 30, 40, and 50 mM glycine (pH 8.50) to achieve pH range for enzymatic reactions, 0.5 mM magnesium chloride, 1 mM sodium citrate, 200 mM sodium chloride, and 15% (vol/vol) glycerol). The optical density of the bacterial samples was adjusted to an OD_600_ of 0.05, then 10 ul of each bacterial sample was used to inoculate a 50 ul reaction containing the given amounts of EC1 proteins in equal mass ratios in reaction buffer. The reactions were then incubated for 18 hours at 37°C. The viability of the cells in the reaction was measured with the LIVE/DEAD BacLight Bacterial Viability Kit (Molecular Probes) via flow cytometry as per manufacturer instructions. Briefly, a 2X working solution of LIVE/DEAD BacLight staining reagent containing SYTO9 and propidium iodide (PI) was mixed with the reactions and incubated for 15 minutes in the dark before measurement. Bacterial populations were gated using Forward and Side Scatter, then the viable population was determined as SYTO9(+) PI(-) and the non-viable populations was determined as SYTO9(+) PI(+).

Liposome Reversal Assay

25mL of *M. abscessus* cells grown to mid-logarithmic phase in 7H9 broth were resuspended in 1mL PBS, then sonicated using the Pixul® sonicator with the pulse set to 25.00, PRF equal to 1.0, process time equal to 15 min, and burst rate set to 1 to create a single cell resuspension of mycobacteria. After sonication, bacterial counts per mL were determined using a BactoBox and diluted to 1.5e7 cells per mL in PBS. Liposome samples were diluted to 8µg of LysB per mL. In a deep well 96-well plate, 160µl of bacterial cells and 200µl of liposome or antibiotic treatment was added to 640µl of 7H9 with or without 0.6% Triton X-100, then incubated at 37°C for 18 hours while shaking. After incubating, 40ul of the reaction was added to 160ul of 7H9 media and grown at 37°C for 24 hours overnight. After growing out, the samples were briefly mixed by pipetting, then the growth measured in a plate reader by OD600.

**Supplemental Methods References**

1. Li W, Godzik A. Cd-hit: a fast program for clustering and comparing large sets of protein or nucleotide sequences. Bioinformatics. 2006 Jul 1;22(13):1658–9.

2. Altschul SF, Madden TL, Schäffer AA, Zhang J, Zhang Z, Miller W, et al. Gapped BLAST and PSI-BLAST: a new generation of protein database search programs. Nucleic Acids Res. 1997 Sep 1;25(17):3389–402.

3. Marchler-Bauer A, Panchenko AR, Shoemaker BA, Thiessen PA, Geer LY, Bryant SH. CDD: a database of conserved domain alignments with links to domain three-dimensional structure. Nucleic Acids Res. 2002 Jan 1;30(1):281–3.

4. Wick RR, Judd LM, Gorrie CL, Holt KE. Unicycler: Resolving bacterial genome assemblies from short and long sequencing reads. PLoS Comput Biol [Internet]. 2017 Jun 1 [cited 2023 Oct 3];13(6). Available from: /pmc/articles/PMC5481147/

5. Wood DE, Lu J, Langmead B. Improved metagenomic analysis with Kraken 2. Genome Biol [Internet]. 2019 Nov 28 [cited 2023 Nov 19];20(1):1–13. Available from: <https://link.springer.com/articles/10.1186/s13059-019-1891-0>
